# Inference of Epidemiological Dynamics Based on Simulated Phylogenies Using Birth-Death and Coalescent Models

**DOI:** 10.1371/journal.pcbi.1003913

**Published:** 2014-11-06

**Authors:** Veronika Boskova, Sebastian Bonhoeffer, Tanja Stadler

**Affiliations:** 1 Department of Biosystems Science & Engineering (D-BSSE), Eidgenössische Technische Hochschule (ETH) Zürich, Basel, Switzerland; 2 Institute of Integrative Biology, Eidgenössische Technische Hochschule (ETH) Zürich, Zürich, Switzerland; Duke University, United States of America

## Abstract

Quantifying epidemiological dynamics is crucial for understanding and forecasting the spread of an epidemic. The coalescent and the birth-death model are used interchangeably to infer epidemiological parameters from the genealogical relationships of the pathogen population under study, which in turn are inferred from the pathogen genetic sequencing data. To compare the performance of these widely applied models, we performed a simulation study. We simulated phylogenetic trees under the constant rate birth-death model and the coalescent model with a deterministic exponentially growing infected population. For each tree, we re-estimated the epidemiological parameters using both a birth-death and a coalescent based method, implemented as an MCMC procedure in BEAST v2.0. In our analyses that estimate the growth rate of an epidemic based on simulated birth-death trees, the point estimates such as the maximum a posteriori/maximum likelihood estimates are not very different. However, the estimates of uncertainty are very different. The birth-death model had a higher coverage than the coalescent model, i.e. contained the true value in the highest posterior density (HPD) interval more often (2–13% vs. 31–75% error). The coverage of the coalescent decreases with decreasing basic reproductive ratio and increasing sampling probability of infecteds. We hypothesize that the biases in the coalescent are due to the assumption of deterministic rather than stochastic population size changes. Both methods performed reasonably well when analyzing trees simulated under the coalescent. The methods can also identify other key epidemiological parameters as long as one of the parameters is fixed to its true value. In summary, when using genetic data to estimate epidemic dynamics, our results suggest that the birth-death method will be less sensitive to population fluctuations of early outbreaks than the coalescent method that assumes a deterministic exponentially growing infected population.

## Author Summary

The control or prediction of an epidemic outbreak requires the quantification of the parameters of transmission and recovery. These parameters can be inferred from phylogenetic relationships among the pathogen strains isolated from infected individuals. The coalescent and the birth-death process are two mathematical models commonly used in such inferences. No benchmark on the performance of these models currently exists. We aimed to objectively compare two specific models, namely the constant rate birth-death model and the coalescent with a deterministic exponentially growing infected population. We compare coverage, accuracy, and precision with which they can capture the true epidemic growth rate parameter using simulated datasets. We find that the constant rate birth-death process can account for early stochasticity and is thus capable of recovering the epidemic growth rates more successfully. Provided one of the parameters is known, e.g. the sampling proportion of infected individuals, then the basic reproductive ratio can also be estimated reliably. We conclude that a birth-death-based method is generally a more reliable method than a deterministic coalescent-based method for epidemiological parameter inference from phylogenies representing epidemic outbreaks. Care should be taken if sampling is not constant through time or across individuals, such scenarios require so-called birth-death skyline models or multi-type birth-death models.

## Introduction

In many applications determining the past dynamics of populations is of interest. In an epidemiological context in particular, the interest lies in knowing two quantities: the basic reproductive ratio 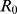 and the growth rate 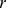 of the epidemic. 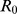 is a key parameter that determines the probability and the extent of spread of the disease in the population. It is defined as the number of secondary infections a single pathogen is expected to cause when introduced into a population of susceptible individuals [1], [2]. The growth rate 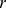 determines the speed of spread of the pathogen. Accurate estimation of these two parameters (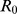 and 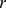) is required in order to take appropriate measures of intervention, e.g. vaccinations or isolation of infected individuals. Until recently, estimation of these parameters was exclusively based on prevalence and incidence epidemiological data. However, recent progress in phylodynamics has enabled the inference of these parameters from pathogen sequence data by integrating methods of phylogenetics with those of mathematical epidemiology (for review see [3]).

SIR-type models have been widely used to describe epidemiological dynamics [1], [4]. In essence, these models are based on separating the population into different classes of individuals, namely susceptibles (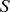), infecteds (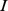) and recovereds (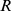). Individuals can change their status, i.e. switch from one class to another. The epidemiological dynamics depend on two rates: a birth rate 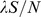 and death rate 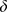, where 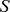 is the number of susceptibles and 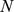 the total population size. The birth rate, or transmission rate, is the rate with which one infected individual will infect another uninfected individual. In the transmission tree, an infection event will be displayed as a bifurcation or split of one lineage into two lineages. The death rate, or removal rate, is the rate with which an infected individual becomes non-infectious, e.g. recovers from the disease, dies, or changes behavior. In the transmission tree, becoming non-infectious is a lineage that stops growing, i.e. becomes a tip in the tree. Various sampling schemes select a proportion of the infected individuals from the complete transmission chain to be included into the observed phylogeny. The observed phylogeny is the subtree induced by the complete transmission chain that connects the sampled individuals. This sub-selection of the individuals reflects the fact that in empirical datasets the pathogens of only a small fraction of the infected hosts have been sequenced and included into an epidemiological study.

For an epidemic following SIR dynamics, the growth of the population size at the initial stage of the spread follows an exponential trend, although it slows down at later stages due to a depletion of susceptibles. We focus on a special scenario, where only the early epidemic outbreak, i.e. exponential growth of the infected population, is being considered. We can simplify the model to a constant rate birth-death process, where we assume no significant decrease in the number of susceptibles over time, formalized as 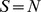, implying that birth rate 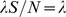 is constant.

Recent genetic sequencing efforts have produced many pathogen sequences from different hosts. To reconstruct their phylogenetic relationships, numerous methods have been developed (refer to books [5], [6] and references therein). The resulting phylogenetic trees are used as a proxy for the (incomplete) transmission tree, and thus provide us with insights into the dynamics of the epidemic. They enable us to estimate parameters such as transmission rate (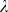), removal rate (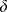), growth rate (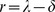), or basic reproductive ratio (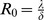). Methods based on Bayesian inference coupled with a Markov chain Monte Carlo (MCMC) procedure [7] infer the posterior distribution of trees (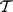) together with the epidemiological parameters (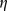) and sequence evolution parameters (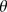) from genetic sequencing data based on the following relation: 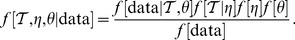
(1)

Here, 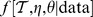 is the posterior distribution of the parameters and trees; 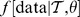 is the likelihood of the parameters (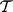 and 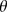) that is usually computed by the Felsenstein algorithm [8]; 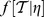 is the probability density of the phylogeny given the epidemiological parameters (e.g. assuming an SIR-type model); 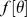 and 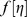 are priors for evolutionary and epidemiological parameters, respectively; and 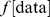 is the normalizing constant representing the integral of the numerator over all phylogenies and parameters. As data are fixed, 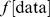 is a constant and thus irrelevant for the estimation of the posterior probability density of the parameters in the MCMC procedure.

Here, we focus on the impact of the underlying epidemiological model when calculating 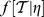. Two models are mostly used in epidemiological contexts for this purpose: the coalescent [7], [9]–[14] (uses and review of the model are described in [15]) and the birth-death process [16]–[25] (reviewed in [26]). Both models have been used to estimate 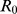 and/or the growth rate parameter 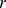 of HCV [27], [28] and HIV epidemics [24], [25], [29].

Since we only focus on phylogenies resulting from early epidemic outbreaks, we apply a special case of the birth-death model, namely the constant rate birth-death model with incomplete sampling, and a special case of the coalescent model, namely the coalescent with deterministic exponential infected population growth. Both models are implemented in the software package BEAST v2.0 [30] and have been used interchangeably for parameter inferences. The constant rate birth-death model implemented for parameter inference in BEAST v2.0 is precisely the epidemic outbreak model introduced above. The specific sampling scheme used in this study is the implementation of a constant sampling probability 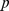 upon “death” (happening with rate 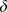) for each individual (in BDSKY add-on of BEAST v2.0), and is known as the incomplete sampling version of the birth-death model [24]. The coalescent with deterministic exponential infected population growth has been introduced in population genetics, and is now also used as an approximation for epidemiological dynamics.

Classically, the coalescent has been used in phylodynamic studies. The coalescent reconstructs the ancestry of 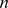 sampled individuals towards the most recent common ancestor (MRCA). In fact, it reconstructs the probabilistic structure of the tree by merging lineages progressively going back in time as a function of the population size until there is only a single lineage left [31]. The coalescent thus provides a prior distribution of trees given a population size, where the population size may change through time. In the epidemiological context the population size of interest is that of the infected individuals. This probability density function allows for the calculation of the probability of the tree for given population size parameters [11], [12], [29]. The coalescent seems to be a good approximation to many processes arising in biology (e.g. [11], [28], [32]). However, violations of the model assumptions can lead to consequences whose nature and extent are still not fully explored [24], [29]. The coalescent can be interpreted as a continuous time approximation of the discrete time Wright-Fisher model [33], [34]. Based on this approximation, as stated by Rodrigo and Felsenstein in [29], the requirements for the studied population to be well approximated by the coalescent are:

1. individuals from one generation give rise to the individuals in the next generation,
2. there exists sufficient genetic diversity within the population to allow reconstruction of the phylogenetic relationships,
3. the population size is large enough (compared to the sample taken), and
4. the population size is small enough to be able to trace back the MRCA.

In most of the coalescent models, a further assumption is made:

1. the population size changes deterministically.

The coalescent can also be interpreted as a continuous time limit of the discrete time Moran process [35]. The assumptions above with exception of 1) are also required for the continuous time approximations of the discrete time Moran model, rather than Wright-Fisher population model. Continuous time versions of Wright-Fisher and Moran population models can also be formulated directly rather than by approximation of discrete time models. This is done by assuming a rate of coalescence in continuous time instead of approximating it by a conversion from discrete to continuous time space, as we point out in the Supplementary Material S1. Such continuous time Wright-Fisher and Moran population models can be formulated as a coalescent process without the assumptions 1) and 3). Furthermore, extensions to avoid deterministic population size changes, i.e. assumption 5), have been developed [36].

In an epidemiological context, the sampling proportion in a recent epidemic can be quite high. For instance, the sampling proportion of the HIV epidemic in Switzerland has been estimated to be 0.75 [37]. This high sampling proportion is a misspecification for the discrete time Wright-Fisher and Moran process-based coalescent models. Although some studies suggest that the violation of this assumption should not be of significant importance [38], its consequences on the model performance when applied to empirical data are so far unknown. In addition, impacts on the parameter inference under the coalescent in the context of deviations from the deterministic population size assumption are not well explored either.

The birth-death model also requires assumptions 2) and 4) to be fulfilled. Additionally, the generation times are assumed to be exponentially distributed, instead of discrete generations as in the assumption 1). We see the major difference between the birth-death model and the coalescent in three factors:

1. The birth-death process produces trees and analyzes phylogenies assuming stochastic population sizes, thus relaxing the deterministic population growth assumption 5) of deterministic coalescent models (note that extensions of the coalescent accommodating stochastic populations sizes have recently also been published [36]).
2. The birth-death process models do not expect the proportion of the sampled individuals in the tree to be small, but rather estimate this quantity along with the other model parameters, i.e. assumption 3) is relaxed.
3. The birth-death process explicitly models the sampling process while the coalescent conditions on sampling, assuming each lineage at a sampling point is equally likely to be the one included. The explicit modelling of sampling is an advantage if the sampling process is known, but a disadvantage if the sampling process is hard to estimate.

In this paper, we want to shed light on the practical importance of the theoretical points raised above for parameter estimation. We investigate the comparative performance of the birth-death and the deterministic coalescent model in phylodynamic parameter estimation by doing a simulation study. We first simulated both constant rate birth-death model trees with incomplete sampling (from now on simply referred to as the birth-death model, unless specified otherwise), and coalescent model trees with deterministic exponential infected population growth (from now on simply referred to as the coalescent model, unless specified otherwise). We then applied phylodynamic methods based on the birth-death model and the coalescent model within the BEAST v2.0 software package to the simulated phylogenies. In this fashion, we estimate the phylodynamic parameters and compare coverage (measured as the fraction of simulated trees where the HPD captures the true parameter), accuracy (measured as the root mean square error (RMSE)) and precision (measured as the width of HPD intervals) of the parameter estimates.

## Results

We simulated 100 trees with 100 tips (for each parameter combination) under the birth-death model at different 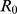, 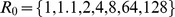. We always assumed that the epidemic was initiated by one single individual. Furthermore, we used a fixed death rate, 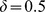 such that the expected time until becoming non-infectious is 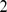 which defines our time unit. We first kept the sampling probability at a fixed value 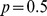, to reveal the effect of 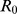 independent of the effect of the sampling intensity 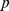. Second, we also used various 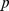 for simulations. We then applied the birth-death model and the coalescent model to the simulated trees to infer the model parameters. The coalescent has parameters growth rate 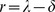 and scaled population size 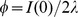 [14], where 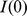 is the present-day infected population size. In addition to the length of the epidemic 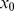, the birth-death model is specified by three population dynamic parameters 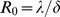, 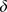, and 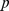. In fact, analog to the coalescent, the birth-death model can only identify two compound parameters, namely 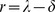 and 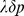 based on phylogenies [22]. As 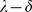 is estimated under both models, we focused on comparing the inferred epidemic growth rate 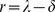.

### Role of stochastic population growth

**Figure 1:**
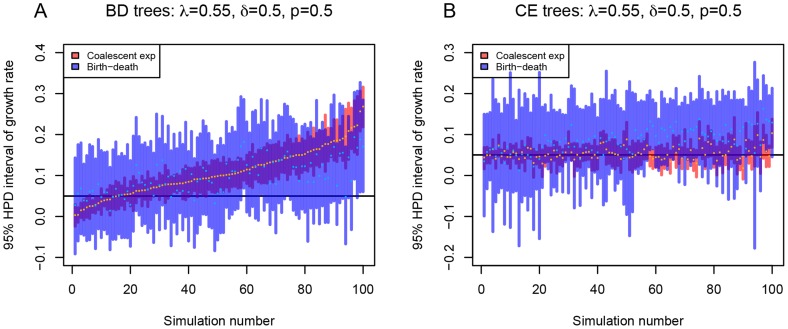
Comparison of the birth-death model and the coalescent model in estimating epidemic growth rate. For each plot, 100 trees simulated under the constant rate birth-death (BD) model with incomplete sampling (subfigure A) or coalescent (CE) model with exponential growth of the infected population (subfigure B) were analyzed assuming a birth-death model (blue bars) or a coalescent model with deterministic exponential population growth (red bars). 95% highest posterior density (HPD) intervals of the growth rate parameter are shown (y-axis). The trees are ordered (x-axis) by the median value of the posterior distribution of the growth rate parameter estimated by the coalescent (orange dot within the red bar) from the birth-death trees. Median of the posterior estimates for the growth rate parameter estimated by birth-death model is indicated as light blue dot within each blue interval. The true value of the growth rate parameter, i.e. the value under which the trees were simulated, is displayed as black horizontal bar. Here, we used 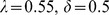 and 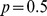 (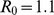). See Figure S1 for the plots of other parameter settings.

The ability of this specific birth-death model and the coalescent model to capture the true growth rate 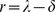 in the 95% highest posterior density (HPD) interval at fixed 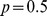 is summarized in Table 1, and shown in Figure 1, Figure S1 and Figure S2. Note that none of these, nor any of the results below, change substantially if we use quantiles instead of HPD intervals (data not shown). For 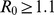, the birth-death model successfully recovers the growth rate parameter for trees simulated under the birth-death model, whereas the coalescent model successfully recovers the growth rate parameter for those trees that were simulated under the coalescent. This observation is not surprising but confirms the basic expectations that the model used for simulation should be good when it is also applied for inference.

**Table 1:** Summary of growth rate parameter estimation statistics.

	birth-death model trees						coalescent model trees					
	birth-death			coalescent			birth-death			coalescent		
	recovered	HPD size	RMSE	recovered	HPD size	RMSE	recovered	HPD size	RMSE	recovered	HPD size	RMSE
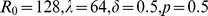	96	0.273	0.072	66	0.257	0.125	98	0.274	0.061	89	0.244	0.079
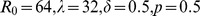	97	0.290	0.072	68	0.255	0.141	97	0.287	0.069	93	0.251	0.070
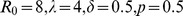	94	0.382	0.093	59	0.324	0.184	99	0.377	0.067	94	0.312	0.087
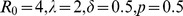	92	0.473	0.126	65	0.358	0.204	99	0.472	0.101	95	0.340	**0.086**
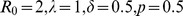	92	0.766	0.224	55	0.432	0.332	97	0.752	0.164	92	0.407	**0.113**
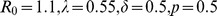	94	4.363	1.278	28	1.440	1.608	90	3.656	0.914	**93**	1.090	**0.335**
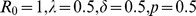	78	Inf	Inf	18	Inf	Inf	57	Inf	Inf	**93**	Inf	Inf

For each of the 100 trees simulated under the respective model (the birth-death or the coalescent), with 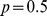 and 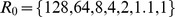, we estimated the coverage by counting the number of times the method applied (the birth-death or the coalescent) estimated a 95% HPD interval containing the true growth rate 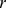 parameter (“recovered” column). The counts in bold indicate instances where the coalescent model has as higher coverage than the birth-death model. Note that both the 95% HPD interval size and the root mean square error (RMSE) value were normalized by the true growth rate (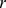) value, such that original size/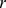 =  normalized size and original RMSE/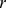 =  normalized RMSE. The RMSE values in bold indicate where the RMSE is lower for the coalescent than for the birth-death model estimates. We display a summary of the medians of HPD sizes across all 100 trees per setting.

In the critical case where 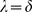, the birth-death model recovers the true growth rate only in 78% of the birth-death trees. This is because the birth-death likelihood is conditioned on the time of origin of the process 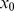 (length of epidemic). Our simulated trees are all of different lengths, however, as we stop once reaching 100 tips. That means that for low growth rates, we select a very biased set of relatively big trees, as most realizations would die out before producing 100 tips. By looking at Figure S1 we observed that especially for low 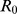 values, i.e. 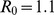 and 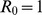, the selective inclusion of the relatively big trees into the final set results in the median estimates of the growth rate parameter to be biased towards higher values than the truth. To show that the birth-death inference method has no bias if applied to trees with a large fixed time of origin, we simulated trees under 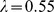, 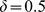, 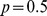 until reaching fixed time 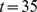, or 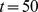 (Figure S3). The 95% HPD intervals of the birth-death model growth rate estimate capture the true value of the growth rate in 95% and 96% of the cases, respectively. The distribution of the medians of the 100 HPD intervals is spread out evenly around the true value of 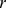, meaning the effect of growth rate over-estimation decreases for increasing 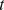.

When applying the birth-death method to coalescent trees, the growth rate coverage is higher than when applying the coalescent method to birth-death trees. The higher coverage of the birth-death model comes partially at the cost of a larger 95% HPD interval size (see Table 1). The normalized 95% HPD interval sizes of 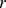 produced by the birth-death process and by the coalescent are almost identical for very large 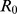 (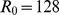), and they increase for both coalescent and birth-death model parameter estimates with decreasing 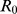. However, the coalescent intervals become smaller than birth-death intervals, and at 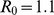 their widths differ by a factor 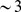. This discrepancy between the HPD sizes does not translate in decreased accuracy of the birth-death model compared to the coalescent model. Accuracy can be measured by the root mean square error (RMSE) of the median of each posterior interval. The accuracy of the birth-death model is higher (RMSE is lower) than the accuracy of the coalescent when applied to the birth-death trees. The accuracy of the birth-death model is lower than the accuracy of the coalescent model on the coalescent trees for our analyses with 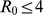. As expected, the above observations do not change when the branch lengths, i.e the time units, are scaled. This corresponds to multiplying the birth and the death rates, and thus the growth rate parameter 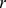, by a constant factor while keeping 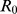 unchanged (Table S1). The increase in HPD interval size when lowering 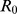 may be due to increasing stochasticity in population size variation over time. This increased stochasticity is caused by decreased population growth resulting from more death events per birth event.

We will now discuss the reason for biases when applying the birth-death method to coalescent trees and vice versa. When applying the birth-death method to coalescent trees, for 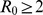, the true growth rate is recovered very reliably. The birth-death process has a small bias when applied to the coalescent trees for 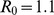 and 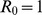. This can be explained again by the simulation scheme and vanishes if simulating for a fixed time rather than until a number of samples is reached (Figure S3).

When applying the coalescent method to birth-death trees, the coalescent misses the true growth rate value (the HPD does not contain the true growth rate) on the birth-death model trees more often than the birth-death model on the coalescent trees. This is accentuated with decreasing 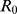. The coalescent has a tendency to overestimate the growth rate (see Figure 1 (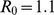) and Figure S1). The growth rate overestimation can be explained by the push-of-the-past effect described by Nee et al [39]. The exponential growth coalescent model assumes a constant population growth rate 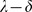. The push-of-the-past effect causes the expected population size under the birth-death model to initially increase faster than with rate 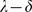, and then slow down to grow with the constant rate 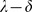. As a consequence, when a final population size is fixed the coalescent trees are predicted to be longer than the birth-death trees. Put in other words, given a fixed time after the start of the process, the expected birth-death population size is bigger than the expected coalescent population size. This push-of-the-past effect becomes more severe for smaller values of 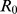. For inference, this means that the coalescent applied to birth-death trees infers inflated growth rates as coalescent trees with the true growth rate would be expected to be longer.

We observe this push-of-the-past effect and the resulting differences in coalescent and birth-death trees in our simulations. We investigated the difference between birth-death and coalescent trees for the parameter combinations where most stochasticity in population size variation over time and most bias of the inferred parameters was observed: 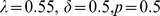, i.e. 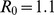. Both the mean and the median measure of the tree lengths without the root-origin distance showed that the coalescent had a strong preference to produce overall longer simulated trees. Median tree length of the coalescent trees was 35.2 while that of birth-death trees was only 26.1. Similarly, the mean tree length was 36.1 and 27.7 for coalescent and birth-death trees, respectively. Upon visual inspection of these trees, we noticed that the coalescent mostly produced trees with longer inner branches, especially close to the root, as compared to trees simulated under the birth-death model.

As the population size growth curves in semi-logarithmic plots are parallel lines with the slope 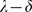 for large 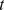, the relative difference between the coalescent and birth-death tree lengths should become smaller for increasing 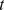. This means that the ratio of the length of the birth-death tree and of the length of the coalescent tree tends to 1 when letting the trees (populations) grow for longer times. In fact, by increasing the final population size through sampling more tips (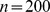, 500 or 1000) and thereby suppressing the importance of the push-of-the-past, the median of the coalescent estimates of the growth rate gets closer to the true value. Nevertheless, the coverage of the coalescent model does not improve due to overconfident estimates, i.e. shrinking HPD intervals (Figure S4).

When applying the birth-death model to the long coalescent trees we occasionally observed an overestimation of the growth rate. In most cases the growth rate is estimated correctly by the birth-death model though. The coalescent trees typically have long branches close to the root. In the following we will demonstrate that these early long branches do not strongly impact the overall likelihood calculation in the birth-death model.

**Figure 2:**
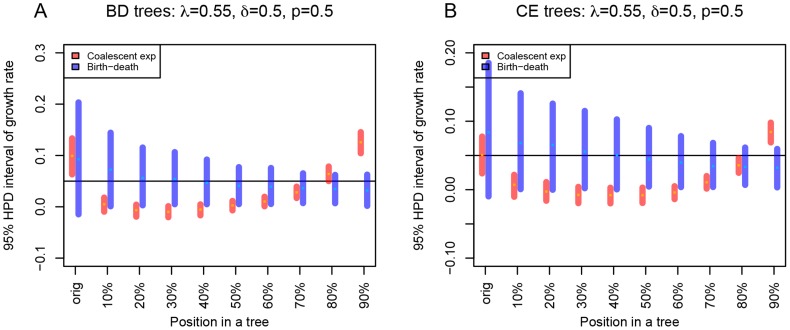
Influence of branch length extension in various parts of the tree on the growth rate parameter estimation. For setting 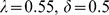 and 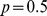 (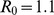), we modified all 100 birth-death trees (A) and all 100 coalescent trees (B) by branch extension. The unchanged tree is denoted as “orig” on x-axis. We added 48 units of time, roughly corresponding to the full length of the longest trees, to the branches. We extended the branches that were present in the tree at 10% of the tree (going from the root), at 20%, 30%, 40%, 50%, 60%, 70%, 80%, 90% (see x-axis from left to right). We then re-estimated the growth rate parameter for each such tree. Unlike in previous plot, here we display a summary in form of the median values of the start and the end of the 95% HPD intervals, and the median of the medians of the posterior estimates for all 100 trees per setting.

To investigate the impact of long early branches on parameter estimation under each model further, we changed the simulated trees systematically. We looked at trees simulated under 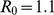 and 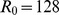. In these trees, we extended each branch existing after 10%, 20%, 30%, 40%, 50%, 60%, 70%, 80%, 90% of the time between the root an the present. We extended branches in all these cases by 48 for 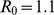 and by 0.18 for 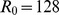, which was approximately the full length of the birth-death tree (Figure 2 and Figure S5).

Re-analyzing the trees with 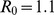 using the birth-death model and the coalescent model revealed high sensitivity of the coalescent estimates to early but not so much to the late perturbations. The birth-death model estimates of the growth rate proved to be more robust to early perturbations than to late perturbations, see Figure 2. We note that the early perturbations actually only affect very few branches, and thus only introduce minor changes to the data. We hypothesize that the ability of the birth-death model to account for stochasticity in population size determines its robustness to above-introduced changes in branching times. When perturbing the tree close to present, the birth-death model growth rate estimates decrease as all branches occurring prior to the perturbation are not allowed to produce sampled descendants later on. For that particular reason, the birth-death method infers a much too low median growth rate when we only extend the single branch leading to the most recent bifurcation (data not shown). Thus, the birth-death method is very sensitive to even minor perturbations close to the present.

Re-analyzing the trees with 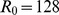 revealed that both the birth-death and the coalescent method are very sensitive to perturbations (Figure S5). This is most likely because the process is very deterministic at high 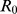 (Figure S2). The perturbation cannot be considered to be the result of stochastic population size changes as at low 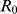, and therefore they significantly influence the overall likelihood values of the inference methods.

Another way to simulate trees under a different model than the birth-death or the coalescent model is to simulate SIS/SIR trees. In both these alternative models, there is an exponential growth of the infected population early on, followed by a decrease in transmissions (births) due to a depletion of susceptibles. In trees produced by an SIS model with small total population size (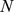), the curve of infected individuals over time follows a logistic trend. In the case of the SIR model assuming a small total population size the growth slows down after the exponential phase, then stops and finally becomes negative, i.e. the population size of infected individuals decreases.

When applying the exponential growth birth-death and coalescent models to the SIS/SIR trees, the birth-death process underestimates the growth rate more severely than the coalescent model in trees which reach the post-exponential growth phase. This fits very well to the results presented above for the constant rate birth-death trees. The birth-death model uses all available information in the tree, and thus obtains an average growth rate estimate from the SIS/SIR trees which is lower than the initial growth rate due to the post-exponential slowdown in transmission. The coalescent mainly uses information from the early epidemic. It thus puts less emphasis on the post-exponential phase and consequently does not severely underestimate the growth rate. The birth-death model does not produce consistently larger HPD intervals than the coalescent (see Table 2), in contrast to the exponential growth trees.

**Table 2:** Summary of growth rate parameter estimation statistics in SIS/SIR trees.

	SIS model trees						SIR model trees					
	birth-death			coalescent			birth-death			coalescent		
	recovered	HPD size	RMSE	recovered	HPD size	RMSE	recovered	HPD size	RMSE	recovered	HPD size	RMSE
N10000: 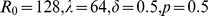	1	0.211	0.293	**63**	0.253	**0.139**	0	0.213	0.294	**59**	0.259	**0.137**
N10000: 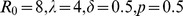	95	0.374	0.101	62	0.320	0.167	96	0.372	0.100	64	0.316	0.174
N10000: 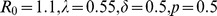	94	4.410	1.262	21	1.374	1.557	91	4.339	1.286	23	1.390	1.743
N500: 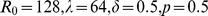	0	0.026	0.949	0	0.013	**0.702**	0	0.021	0.967	0	0.010	**0.769**
N500: 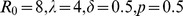	0	0.251	0.442	**77**	0.292	**0.119**	0	0.222	0.562	**73**	0.288	**0.135**
N500: 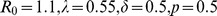	97	4.251	0.904	44	1.276	1.235	99	3.848	0.586	50	1.264	1.133
N300: 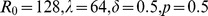	0	0.016	0.971	0	0.009	**0.824**	0	0.010	0.988	0	0.005	**0.896**
N300: 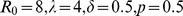	0	0.194	0.617	**59**	0.269	**0.143**	0	0.145	0.799	**70**	0.263	**0.128**
N300: 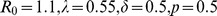	98	4.240	0.806	46	1.242	1.121	100	3.662	0.630	47	1.355	1.162

For each of the 100 trees simulated with the respective method (SIS or SIR model) and the total population size N of 300, 500 or 10000, we estimated the coverage, the 95% HPD interval sizes and RMSE of 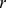 by the birth-death model and the coalescent model, and display the summary of these measures.

In summary, the coalescent model estimates of the growth rate seem to be influenced most strongly by the early branching patterns in the tree. These early patterns most strongly reflect stochasticity in population size. In contrast, the birth-death method averages the information throughout the tree.

### Role of sampling probability

Since the sampling probability is fixed to quite a high value (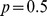) in all the trees simulated above, the trees are not only relatively short, but also a lower 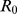 translates to more death events per birth event, and consequently means higher sampling from the population (as we sample from the individuals that become non-infectious with sampling probability 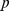). We further investigated if this relatively high sampling causes the coalescent methods to fail for low 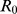.

We simulated trees at low, medium and high 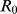, 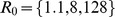, but this time also at different sampling probabilities 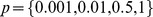 (see Table 3). For some parameter settings with very low sampling probability, the tree simulations did not finish within 7 days of simulation, and the results are thus not displayed in the summary table.

**Table 3:** Summary of growth rate parameter estimation statistics at various 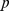.

	birth-death model trees						coalescent model trees					
	birth-death			coalescent			birth-death			coalescent		
	recovered	HPD size	RMSE	recovered	HPD size	RMSE	recovered	HPD size	RMSE	recovered	HPD size	RMSE
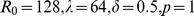	87	0.284	0.085	62	0.264	0.144	98	0.286	0.065	91	0.251	0.080
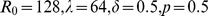	96	0.273	0.072	66	0.258	0.125	98	0.274	0.061	89	0.244	0.079
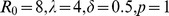	93	0.442	0.113	68	0.348	0.185	100	0.438	0.098	91	0.331	0.104
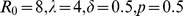	94	0.382	0.093	59	0.324	0.184	99	0.377	0.067	94	0.312	0.087
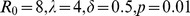	98	0.267	0.066	69	0.246	0.115	98	0.261	0.058	91	0.238	0.074
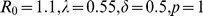	89	6.244	2.088	25	1.858	2.400	86	4.716	1.407	**94**	1.416	**0.420**
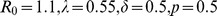	94	4.363	1.278	28	1.440	1.608	90	3.656	0.914	**93**	1.090	**0.335**
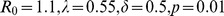	96	0.787	0.196	55	0.395	0.287	98	0.776	0.150	94	0.394	**0.111**
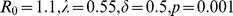	88	0.404	0.121	63	0.326	0.190	97	0.404	0.097	93	0.304	**0.088**

For each of the 100 trees simulated under the respective model (the birth-death or the coalescent), with 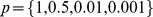 across each 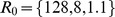, we estimated the coverage, 95% HPD interval sizes and RMSE of 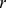 by the birth-death model and the coalescent model, and display the summary of these measures. Simulations under some parameter combinations did not finish within a week, and are thus omitted from the table.

We observed that the size of the 95% HPD interval become smaller with lower sampling probability for both birth-death and coalescent estimates of the growth rate. This means that both methods become increasingly confident in the growth rate estimates with increasing tree length due to decreased sampling.

Additionally, the smaller the sampling probability in the birth-death tree simulation, the more often the true growth rate parameter is recovered by the coalescent. The same is observed for the growth rate parameter estimate produced by the birth-death model on the coalescent trees.

In fact there are two ways to grow the tree longer, either by decreasing the sampling probability or by sampling more tips. As discussed in the previous section, growing the tree longer decreases the push-of-the-past effect. In contrast to decreasing the sampling probability, the coverage of the coalescent method does not improve when sampling more tips. This is presumably because stochastic effects are not diluted when sampling densely. This is best seen when comparing the coverage of the coalescent on birth-death trees grown for longer by increasing the final sample size (as in Figure S4) to that on the trees grown longer by decreasing the sampling probability (Table 3). An especially informative comparison at 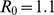 can be made between trees where the final sample is equal to 10000, the average tree length of which (root-origin distance not included) is 105.9, and the coverage is 8/100 (figure not shown) and those trees grown with 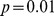, reaching average length of 99.7 and coverage of 55/100.

Overall, the coalescent struggles most with correct growth rate estimation for datasets with low 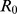 and high sampling probability. At low 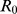, compared to high 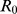, we have a strong push-of-the-past effect and remain for longer in the phase of strong stochastic changes in population size over time. A high sampling probability means that most samples are taken in the early phases of the epidemic, the phase with the push-of-the-past effect and reflecting stochasticity in population size the most.

It could be argued that a high sampling probability which leads to a high sampling proportion is a violation of one of the main assumptions of the coalescent model and the main reason for the biases of the coalescent when applied to birth-death trees. Indeed, for the discrete time Wright-Fisher and Moran model, we have to assume a small sampling proportion when deriving the continuous time coalescent approximation. However, as we show in the Supplementary Material S1, the coalescent can also be interpreted as a continuous time Wright-Fisher and Moran model, and these models do not require a small sampling proportion. In fact, one can even assume complete sampling, i.e. 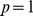. Therefore, we suspect that the high sampling proportion just unmasks the real reason for the frequent inability of the coalescent method to include the true value of the growth rate parameter of the birth-death trees in the 95% HPD interval. The real reason being the stochastic population size variation over time.

**Figure 3:**
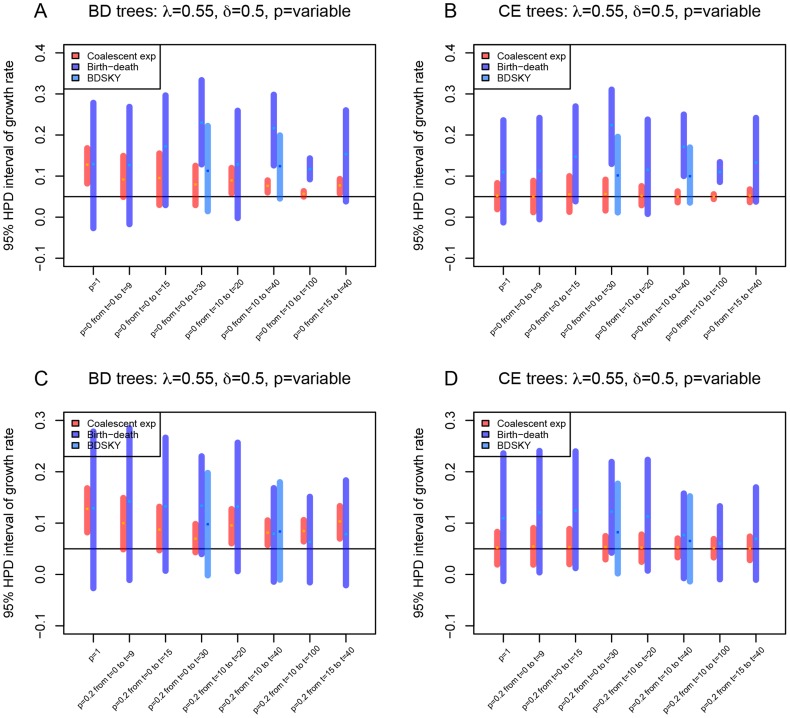
Influence of sampling scheme on the growth rate parameter estimation. For setting 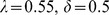 (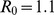), we modified the birth-death tree simulations to include periods of higher (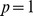) and lower sampling (either 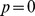, subfigures A and B, or 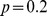, subfigures C and D). We simulated 100 birth-death trees (A and C) and corresponding coalescent trees (B and D) under various sampling schemes (see x-axis annotation). We display a summary in form of the median values of the start and the end of the 95% HPD intervals, and the median of the medians of the posterior estimates for all 100 trees per setting. For the settings where the constant rate birth-death method produced very severe biases, we also analysed the trees with the birth-death skyline model with 10 intervals for the sampling probability (BDSKY, light-blue lines). The summary for trees simulated under constant sampling 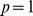 throughout, is represented on the very left of each figure (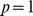 on the x-axis). Next, we varied the sampling as to e.g. sample no tips (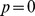) in the early phases (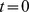 until 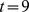) when going forward in time and then sampling all the tips that die (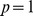) from 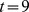 onward (corresponding to the setting denoted as “p = 0 from t = 0 to t = 9”).

Finally, we investigated the sensitivity of the models towards variation in sampling schemes. We simulated trees where periods of no, 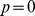 (or low, 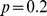), sampling at the beginning were followed by a period of complete sampling, 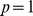. Furthermore, we simulated trees where a period of initial complete sampling was followed by a period of no (or low) sampling, and later again followed by a period of complete sampling (Figure 3 and Figure S6). The coalescent model is very robust to these changes in sampling schemes. The birth-death model is robust to slight sampling variations, but overestimates growth rate severely for extreme changes to the sampling scheme in particular for high 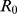. Use of the birth-death skyline model, assuming a time-varying sampling probability rather than a constant sampling probability, reduces this bias (Figure 3 and Figure S6, Table S2).

### Inference of epidemiological parameters beyond growth rate

So far we only investigated the inference of epidemic growth rate using the birth-death and the coalescent models. Both models also estimate other epidemiological parameters. The birth-death model is parameterized by the transmission rate 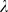, becoming-non-infectious rate 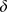 and the sampling probability 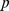. The coalescent model is parameterized by 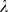, 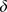 and 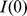. These parameters as well as the compound parameter 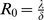 can be inferred given we fix one of the three model-specific parameters.

For the birth-death process, so far we fixed 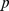 to the true value during the analyses. Now we investigate to what extent we can estimate the individual parameters, including 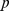, using the birth-death method. We re-analyzed all birth-death and coalescent trees simulated above applying the birth-death model estimating 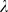, 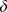 and setting 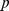 to 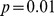, 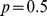, 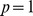 (and/or 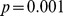, if this was used for tree simulation), and not fixed sampling probability but assume a uniform prior over interval 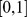. For example, trees produced under 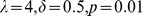 were analyzed under 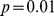 (true 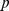), 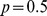, 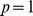 and 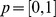.

The likelihood of a tree only depends on 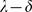 and 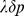, rather than on three parameters 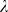, 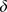, 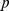 [25]. We could confirm that no matter what 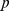 is used for the analysis, true growth rate 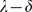 is equally well estimated by the birth-death process for both stochastic birth-death trees or coalescent trees (Figure S7 shows results for trees simulated under 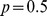). The same holds for estimation of 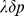 (Figure S8 displays the results for trees simulated under 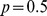).

During this analysis, we also noticed when we set 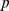 to its true value (i.e. the value used during the tree simulation), we are able to recover the true 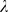 and 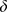 parameters, and consequently also the true 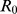 from both the trees generated under the birth-death model and those generated under the coalescent model (see Figures S9, S10, S11 and S12).

**Figure 4:**
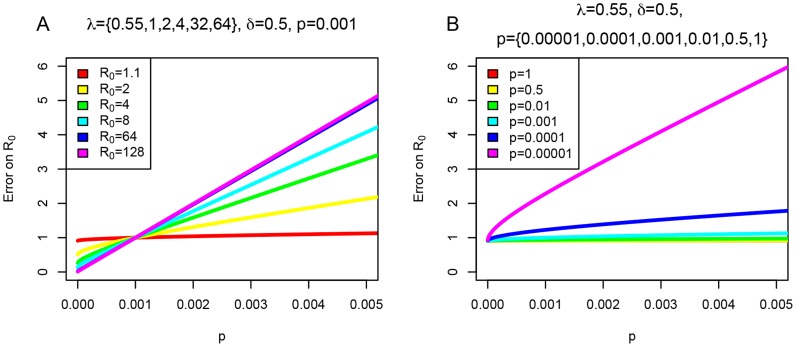
Error on 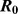 as a function of sampling probability 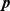 for fixed 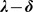 and 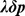. In (A) the relationship between the error on 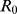, i.e. estimated 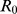/true 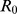, and the sampling probability 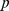 is plotted. The values 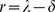 and 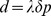 are fixed. For different 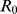, 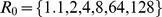, and 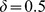 and 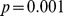, we calculate 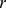 and 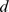, and plot the impact on 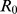 error when changing 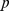 during inference using Equation (3) in the Supplementary Material S1. In (B) we display how error on 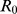 depends on different assumptions of 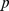 during inference for 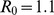, and 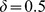 and an array of true sampling probability 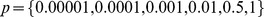 used for calculating 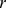 and 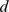.

In the Supplementary Material S1, Section “Parameter correlations under the birth-death process”, we show analytically that for fixed 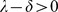 and 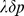, 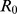 increases, and 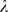 and 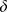 decrease with increasing 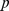, and vice versa. In Figure 4, we plot the impact of changing 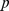 on the 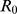 value. We confirmed this theoretically predicted bias in parameter estimation in our simulation study. If we fixed 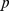 during the birth-death analysis to a bigger value than the true 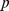 used during the birth-death simulations, then we overestimated 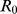 and underestimated 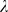 and 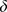, and vice versa. Similarly, when analyzing the coalescent trees with the birth-death model, we observed an upward shift for 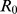 and downward shift for 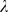 and 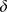 when assuming a 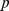 value bigger than used in the simulation of the sampling times, and vice versa.

Using a uniform prior for 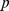 over the interval 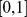 had different effects on estimation of 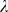, 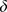 and 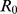, depending on the sampling probability 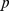 used for simulation. First, in cases where the true 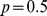, use of uniform prior for 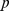 during the analysis resulted in wider 95% HPD intervals that either fully, or mostly, contained the 95% HPD interval produced when 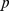 was fixed to the true value (Figures S7, S8, S9, S10, S11 and S12). This is because the value 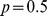 is the median of the prior. Second, for simulated trees with a true 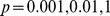, the 95% HPD intervals produced using a uniform prior on 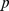 were shifted away from the 95% HPD intervals that resulted from analysis where 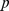 was fixed to the true value (data not shown). As predicted by derivations in the Supplementary Material S1, for a true 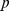 below 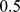, the estimated interval for 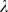 and 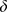 was shifted downward, compared to the interval estimated when the 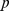 was fixed to the true value, and the estimated interval for 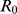 was shifted upward. When the true 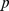 used for simulations was higher than 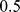, the posterior intervals for 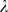 and 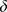 shifted upwards, whereas the posterior interval for 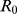 shifted downwards.

**Figure 5:**
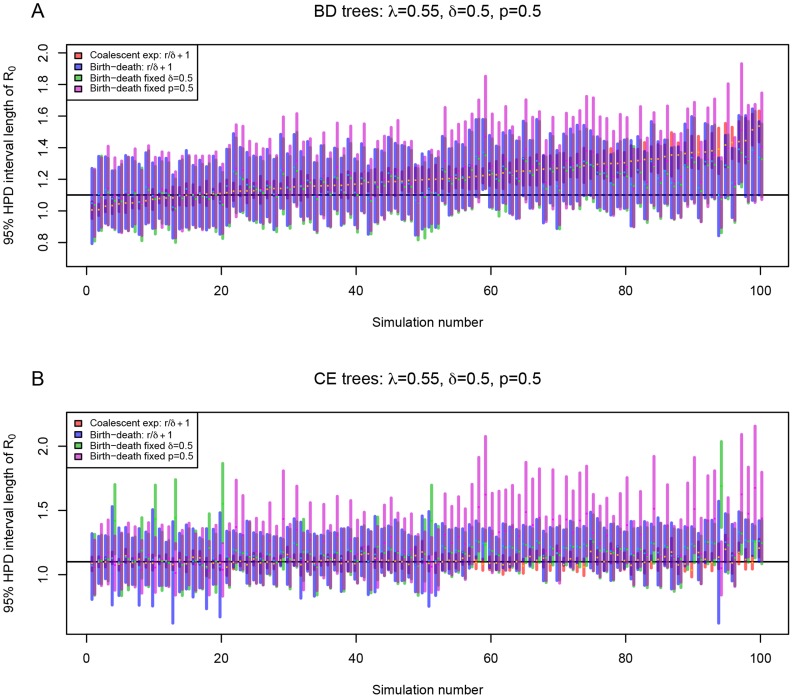
Effect of different information used in the 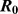 parameter inference. For setting 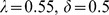 and 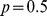 (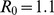), we estimated the 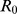 parameter from the birth-death trees (A) and the coalescent trees (B) using four methods. First, using the coalescent posterior estimates of the growth rate 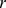 and the true 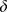, we obtained 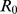 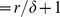 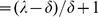 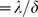 (red bars). Second, we used the birth-death posterior estimates of 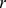 (trees analysed under uniform priors for 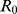, 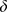, and 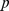), and the true 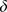 in the post-processing (blue bars), similar to the procedure used for the coalescent. Third, we also analyzed the trees by fixing the prior on the death rate 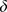 to the true value, 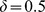 (green bars) or by fixing the prior on the sampling probability 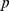 to the true value, 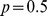 (purple bars) during the MCMC analysis. Note that y-axis now displays 95% HPD of the 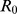 parameter, and within each figure, the trees (simulations) are ordered (x-axis) by the median estimate of growth rate 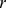 parameter estimated by the coalescent on the birth-death trees.

Overall, the birth-death method recovers two out of the three individual epidemiological parameters 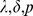 reliably if one of these parameters is fixed (here 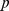). The epidemic growth rate 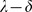 can be recovered well even if 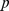 is misspecified. If 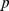, or any of the other two parameters, 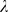 or 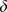, is set to the true value, we can recover 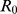 (Figures 5, S13 and S14, for fixed 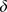). If any of the parameters is fixed to a wrong value, e.g. if one assumes incorrect 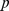, then the original (true) 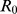 cannot be recovered.

Equivalently, when using the coalescent for inference, and knowing one of the parameters 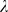, 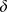 or present day infected population size 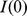, we can also recover the 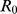 parameter, given we estimated the growth rate 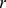 correctly (Figure 5 and Figure S14 display the scenario where 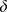 is known). We use the transformation 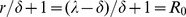 [40] to obtain 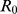 estimates from the posterior estimates of the growth rates.

### Sampling tips at one point in time

**Figure 6:**
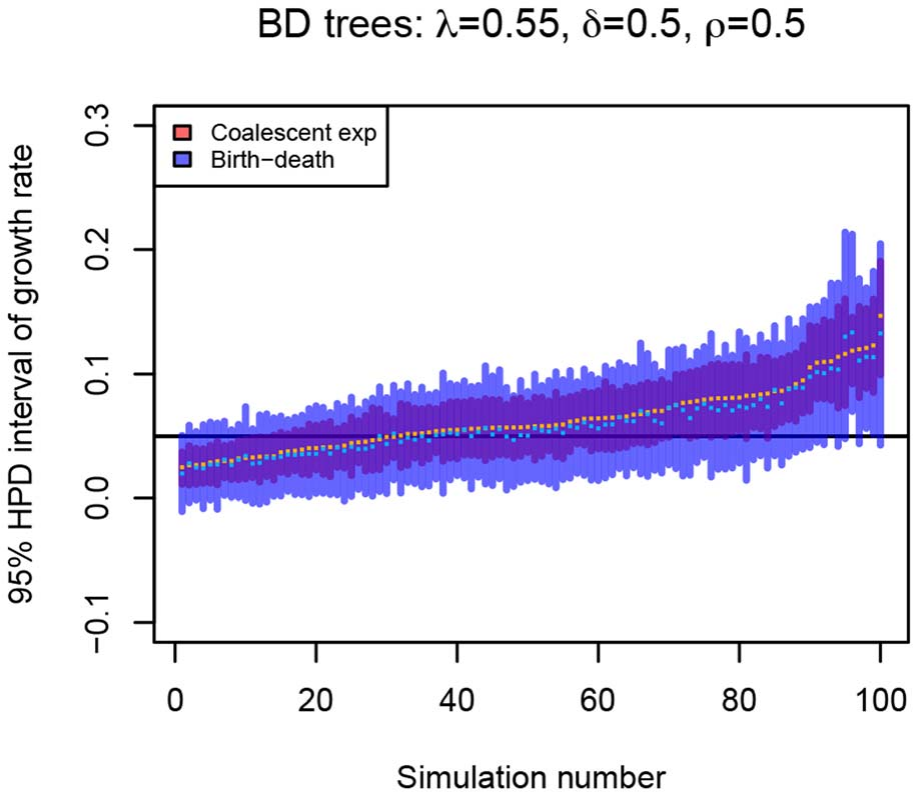
Comparison of the birth-death model and the coalescent model in estimating epidemic growth rate from trees with tips sampled at one point in time. For simulated trees where all 100 tips are sampled at one point in time, we estimated the growth rate parameter assuming a birth-death model with fixed sampling probability 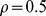 (blue bars) and the coalescent model with a deterministic exponentially growing population (red bars). Here we used 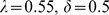 and sampling probability 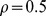 (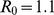). See Figure S15 for the plots of other parameter settings.

The birth-death inference method is partially informed by the sampling times, as the sampling times are outcomes of the birth-death process with constant rate sampling. The coalescent is only informed by the branching times in the tree, as the coalescent conditions on sampling. In order to compare the ability of the coalescent and the birth-death model to infer the underlying population dynamic parameters in the absence of information on sequentially sampled tips, we simulated 100 trees on 100 tips sampled at one point in time under the birth-death model. The simulation was done with ‘TreeSim v2.0’ on CRAN [41] (function sim.bd.taxa). We assumed the two extremes, 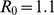 and 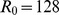 with 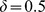. We assumed a uniform prior 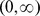 on the length of the epidemic 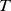 and sampled each infected individual at time 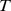 with sampling probability 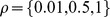. For parameter inference, we again used the birth-death model in BEAST v2.0. As the choice of 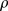 during inference does not influence the growth rate estimate [22], analog to fixing 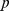 in the sequential sampling model not influencing the growth rate estimate, we fixed 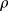 to the truth. For the birth-death model, the true growth rate was contained in the 95% HPD intervals in 91–96 out of 100 cases. For the coalescent, the true growth rate was contained in the 95% HPD interval in 63–75 out of 100 cases. The increased coverage of the coalescent model compared to the sequential model is likely due to two factors: 1) the trees are in general longer meaning the early stochastic fluctuations are less dominant, and 2) there is no sampled data during the early very stochastic changes in population size, as all samples are collected only at the end of the epidemic. The coalescent still does not reach more than 80% coverage because similar to the case of sequential sampling, too narrow HPD intervals are being inferred, see Table S3, and Figures 6, S15 and S16. Overall, when using phylogenies generated under the birth-death process, the birth-death model produces better results than the coalescent even in the absence of sequential sampling time information. Thus, when analyzing sequentially sampled phylogenies, the better performance of the birth-death model does not exclusively come from using the information of the tip times explicitly. This supports our claim that the main reason for the birth-death model in general producing more reliable estimates comes from it taking stochastic population size changes explicitly into account.

### Point estimate of the epidemic growth parameter

**Figure 7:**
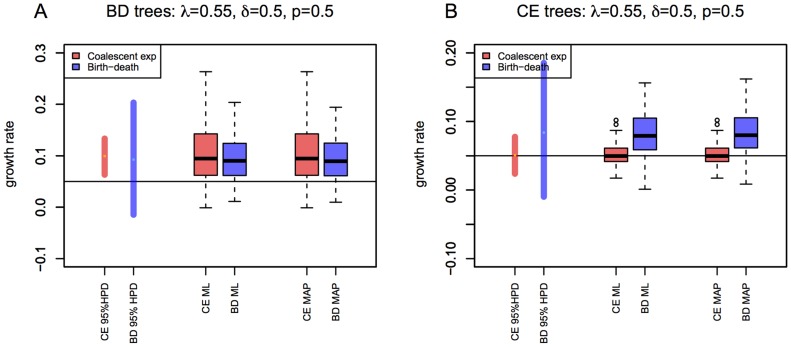
Comparison of growth rate point estimates of the birth-death model and the coalescent model. For setting 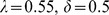 and 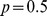 (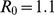), we display the ML and MAP estimates for the birth-death trees (A) and the coalescent trees (B). As a comparison, the median values of the start and the end of the 95% HPD intervals, and the median of the medians of the posterior estimates for all 100 trees per setting are also displayed. The true value of the growth rate parameter, i.e. the value under which the trees were simulated, is displayed as a black horizontal bar. See Figures S17 and S18 for the plots of other parameter settings.

As seen above, the coalescent is often overconfident, i.e. it estimates too narrow 95% HPD intervals, and thus often does not contain the true growth rate parameter. For comparison, we calculated point estimates both for trees with tips sampled sequentially and for trees with tips sampled at one point in time. For various parameter combinations we determined the maximum a posteriori (MAP) estimate from the final MCMC runs. We also report the maximum of the tree likelihood values (ML) of the final MCMC run. For uniform priors and fixed time to origin 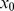, the ML corresponds to the maximum likelihood estimate. We verified this for trees with sequentially sampled tips by estimating the maximum likelihood values with ‘TreePar v3.0’ on CRAN [42]. In contrast to the findings obtained when using the HPD intervals, Figures 7, S17, S18 and S16 show that the 100 ML and 100 MAP point estimates on the birth-death trees do not differ much for the coalescent and the birth-death inference method. Similarly to the results obtained when using HPD intervals for the coalescent trees, the birth-death model overestimates growth rates for high 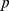 and 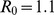 and 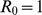. This highlights that when evaluating the performance of different inference methods, we should always consider the HPD/credible/confidence intervals in addition to the point estimates.

## Discussion

Under both the birth-death and the coalescent models, the times of coalescence or bifurcation are stochastically selected from the distribution of coalescence and bifurcation event times. The individual trees produced by both the coalescent and the birth-death process are thus stochastic realizations of the respective processes. However, the coalescent model with exponential growth of the infected population assumes deterministic changes in the population size. Therefore, the trajectory of the infected population size follows an exponential growth. It has been pointed out before [31] that the coalescent model can appropriately approximate population dynamics reflecting models where the sampled genealogy is conditioned on the total population size that varies deterministically.

It has been postulated earlier [29] that coalescent approximations are good for datasets where the sample size 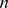 is sufficiently smaller than the population size 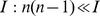. On one hand, this approximation is necessary when translating the discrete time Wright-Fisher and Moran model population dynamics into a continuous time coalescent framework. In fact, such an inequality should hold also when 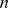 is the number of co-existing lineages in the tree at any time point in the process. On the other hand, the assumption of small sampling proportion (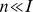) is not required when the coalescent is interpreted as a continuous time Wright-Fisher and Moran model, and has been found unimportant for some inferences based on the Kingman coalescent [38].

We show that the ability of the coalescent with deterministic exponentially growing infected population to properly estimate the dynamical parameters of the birth-death trees derived from early epidemics is questionable, which was previously suggested by the small simulation study in [24]. It often struggles with, and overestimates the growth rate parameter from the birth-death trees representing samples from early epidemics starting with one single infected individual. This effect is accentuated when decreasing the basic reproductive ratio 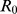 and increasing the sampling probability 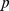. These settings (small 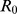 and high 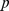) produce birth-death trees that display a significant push-of-the-past effect, and thus differ in their length (age) the most from the coalescent model trees. Furthermore, both increasing the sampling probability and decreasing 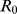 lead to smaller infected population sizes while samples are collected, which in turn corresponds to more stochasticity in population size changes over time reflected in the final trees. Thus, we hypothesize that the coalescent model with deterministic exponentially growing population fails to recover population dynamic parameters when stochasticity is important.

This claim is supported by three observations. First, the birth-death trees show systematic difference from the coalescent trees in their lengths due to the push-of-the-past effect. This effect is the strongest in low 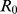 regimes, the regime where we observe that the coalescent struggles most with parameter recovery. Second, early stochastic effects in the population size changes are not well captured by the coalescent. This became apparent when we introduced artificial perturbations to branching times early in the tree, which had a large effect on parameter estimates. In fact, the early phase in the tree informs the coalescent parameters the most, and late perturbations in the tree have little impact on parameter estimates. Additionally, increasing the sampling probability, especially at medium and low 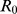, where the population growth in the constant rate birth-death model displays much stochasticity, results in the coalescent failing most often. Even if we increase the final infected population size by sampling more tips, i.e. sampling for a longer time period, this effect is not corrected for and the coalescent has a very low coverage. Third, the coalescent does not capture the true growth rates in many cases because the HPD intervals are narrow around the median estimate. Short HPD intervals likely result from the coalescent model only considering one population size trajectory, namely the deterministically growing exponential population size, per epidemiological parameter combination and ignoring any stochastic uncertainty. The obvious solution to these problems would be to use the coalescent that accounts for stochastic population sizes as in Rasmussen et al. [36]. It is expected that such a model would show an increased coverage rate, but also increased HPD interval sizes, similar to those produced by the birth-death model. It would also be interesting to investigate the performance of the two models on the trees resulting from the epidemics starting with more than a single individual.

In contrast to the coalescent, stochasticity in population size is incorporated into the birth-death process. The birth-death model performs well not only on birth-death trees but also on most coalescent trees. This is due to the birth-death process interpreting variation in branching times as stochastic changes in population size over time leading to large HPD intervals. The birth-death model therefore accepts many different parameter combinations that may have given rise to the observed tree. Furthermore, the ability of the birth-death model to correctly estimate the parameters from the coalescent trees decreased only in those trees that were simulated under combination of high 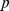 and low 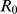. However, unlike in the case of the deterministic coalescent model, in this case the bias can be easily corrected for by letting the trees grow for longer, by sampling more tips or by decreasing the sampling probability. It could be argued that the superior performance of the birth-death model in terms of coverage of the true growth rate comes from explicit usage of the sequential tip sampling times. As we have shown here, even in the absence of information from the sequentially sampled tip times, the birth-death model shows higher coverage than the deterministic coalescent model. This means that it is not only the sampling process that improves the coverage of the birth-death model, but the branching time information alone already leads to better estimates under the birth-death model compared to the deterministic coalescent.

The birth-death model is sensitive if large parts of the tree depart from the birth-death model assumption, as the birth-death model averages over observations throughout the whole tree. This sensitivity becomes particularly apparent in the analysis of SIS/SIR trees and of trees with varying sampling probability over time. Both the birth-death skyline [25] and coalescent skyline plot [28] method aim to capture transmission and removal rate changes over time. Furthermore, the birth-death skyline plot accounts for the sampling probability to change throughout the tree. If the sampling probability changes frequently, it might become hard to obtain good estimates for it. The coalescent conditions on the sampling times and hence does not face the problem of estimating highly varying sampling probabilities. It however still assumes that at the time of sampling, a lineage is chosen uniformly at random from all co-existing lineages. Developing a birth-death based inference tool conditioning on sampling might make use of the advantages of the birth-death and the coalescent tools: accurate inference of HPD intervals by the birth-death method and robustness to time-heterogeneous sampling by the coalescent. In case of lineage-specific sampling, multi-type birth-death models can be employed [43].

The differences of the birth-death method and the coalescent method are mainly observed in the 95% HPD intervals, while only minor differences were apparent in the point estimates, i.e. the maximum a posteriori and maximum likelihood estimates. When comparing the performance of inference methods, it is therefore crucial to always assess the capability of recovering the uncertainty in parameter estimates.

While our study only compared the birth-death and the coalescent methods on simulated trees, previous work also compared the two models on empirical data. A higher estimate of the growth rate parameter of the HCV epidemic in Egypt was reported previously when data were analyzed with the coalescent model as compared to the birth-death model [22]. Furthermore, the coalescent posterior for growth rate displayed a larger spread (standard deviation) compared to the birth-death model. The larger standard deviation should indicate larger HPD intervals. Similarly, in [24], a higher growth rate estimate with larger 95% HPD interval size was observed for Swiss HIV under the coalescent compared to the birth-death model. As for our birth-death simulated trees, the HPD intervals obtained using the coalescent model compared to the birth-death model are shorter, the results based on empirical data contradict these simulation results. However, results from both of these studies are consistent with the epidemic following either the SIS or the SIR-type dynamic, already reaching the post-exponential growth phase (compare with our simulation results in Table 2). Indeed, application of the SIS model to the HIV epidemic in Switzerland rejects the simple birth-death model and reveals a higher 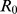 [44] than previously estimated [24].

In the present study we estimate epidemiological parameters based on fixed trees. We note that the tree probability only depends on the branching (transmission) and removal times. Thus, the Bayesian estimates of growth rate 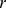 from trees under full sampling, i.e. 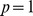, are equivalent to estimating the posterior growth rate from full incidence and prevalence data.

Birth-death based methods explicitly parameterize epidemiological parameters beyond the growth rate 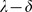. Previously reported correlations when estimating 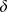, 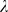, and 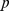 simultaneously [22], [25] are confirmed by our simulation results. In fact, we can only estimate two compound parameters, namely growth rate 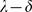 and 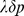, when we have no information beyond the phylogeny. If we are only interested in these two parameters, we can fix one of the tree individual parameters, e.g. sampling probability 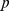, to an arbitrary value. Caution must be taken, however, when drawing any conclusions on 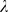, 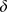, 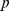 and 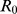. Fixing one of these parameters during the data analysis to a wrong value will produce biased estimates of the remaining parameters. Similarly, 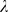, 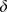, 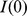 and 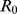 can be estimated by the coalescent model, given that one of these parameters is known, and conditioned on the fact that the growth rate 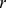 and scaled population size 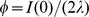 were inferred correctly.

In general, when one knows what kind of population dynamic process gave rise to the tree, the appropriate method should be applied for phylodynamic parameter estimation based on such trees. In case of doubt of the underlying process generating the genealogy, our results show that in scenarios of constant sampling and exponential population growth, especially when the samples were drawn early in the epidemic outbreak, the constant rate birth-death process with incomplete sampling is a better choice than the coalescent assuming deterministic exponential infected population growth.

The assumption of exponential spread of a pathogen with constant sampling is limiting since many epidemics are better characterized by SIS or SIR dynamics with time-varying sampling, for example. Recent work proposed birth-death based SIS and SIR models [44], [45] with time-varying sampling [25], and coalescent based SIS and SIR models [13], [14] for phylodynamic inference. It is not clear how important stochasticity is for the epidemiological SIS/SIR models. Future work should thus focus on a detailed comparison of birth-death based and coalescent based SIS and SIR models, such that the empirical data can be analyzed and interpreted using appropriate methods.

## Materials and Methods

### Birth-death tree simulations

We used three epidemiological SIR-type models to simulate transmission trees growing forward in time, namely the constant rate birth-death process (epidemic outbreak), the SIS model and the SIR model. All of the models are implemented as a Gillespie algorithm [46] in the R package ‘TreeSim v2.0’ on CRAN [41] in the function sim.bdsky.stt.

All models have a common birth or transmission rate 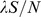 with which one infected individual transmits the pathogen, where 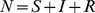 is the total population size assumed to be constant. In the birth-death model, the impact of the susceptible population size is assumed to be neglected and thus 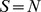 meaning transmission rate 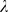 is constant. In all models, infected individuals become non-infectious with rate 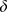. In the SIS model, a recovered (removed/non-infectious) individual goes back into the susceptible class, while in the SIR model, a recovered individual goes into the recovered class. Note that in the constant rate birth-death model, the fate of a recovered individual does not have to be modelled, as the number of susceptibles does not influence transmission rate.

These models induce transmission trees in the following way. Each infected individual is represented by a lineage. A transmission event results in a branching event, while a removal event results in the termination of a lineage, thus a tip in the tree. We assume that we sample each tip from the complete transmission tree with probability 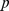, acknowledging that in empirical data only a fraction 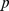 of the infected individuals are sampled and thus included into the dataset. The tree on the sampled tips is called the (observed) phylogeny, on which we do all our analyses. Note that the sampled tips are spaced through time, i.e. serially sampled.

Initially, the population size of infected individuals simulated by these models is increasing at the rate 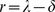. Thus, in expectation, the total infected population size at time 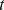 before present time 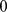 is 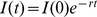.

The constant rate birth-death process always stays in the exponential growth phase. Under the SIS model, we have a finite total population size 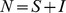, resulting in expectation in a logistic curve of number of infecteds over time: the initial exponential growth phase is followed by the slow down (saturation) of the apparent growth rate of the epidemic until the equilibrium 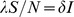 is reached and the growth ceases.

Under the SIR model, there is only a single one-way flow of individuals from 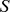 to 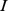 to 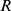 and those that recover cannot become susceptible again, nor are they replaced by new susceptible individuals. The total population size is again limited and constant, 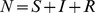. Given the definition of the model, the number of infecteds over time first increases exponentially, then slows down (when there are still susceptible individuals available) reaching a peak, and then declines to 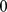. The number of susceptibles in turn constantly decreases, and the number of recovered individuals constantly increases.

For each chosen parameter setting of 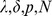, we simulate 100 trees. We stop each tree simulation once we reach 100 serially sampled tips.

### Coalescent tree simulations

We produce trees generated both by SIR-type models (constant rate birth-death, SIS and SIR models above) and by coalescent models for optimal comparison of performance of the birth-death and the coalescent model in estimation of phylodynamic parameters.

We used the BEAST v2.0 package [30] to simulate coalescent trees with temporarily spaced leaves by sampling from the prior distribution of trees generated by the coalescent model with deterministic exponentially growing population (example xml is in File S1). For the coalescent simulation, we used the same parameter settings as in the birth-death simulations whenever possible. Thus, we used 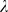 and 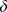 to specify the rate of exponential population growth 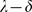 (as under the birth-death model the population size also grows exponentially in expectation), as well as generation time (inter-transmission interval length  = 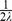 [14]). As the coalescent does not model a sampling process, we conditioned the sampling times for each of the 100 coalescent simulations to those obtained by the birth-death simulation. Further, we assumed the present day infected population size to match the final infected population size in the birth-death simulation. During each coalescent tree simulation, we sampled 10,000,000 trees from the prior and chose the last one (10,000,000th) to be analyzed by the birth-death model and the coalescent model in order to infer the growth rate parameter.

### Parameter inference based on simulated datasets

The constant rate birth-death model with incomplete sampling and the coalescent model with deterministic exponential population growth were applied to the simulated trees to infer the posterior distribution of parameters. For this purpose, we used equation (1). As we did all analyses on fixed trees and did not use sequence data, only 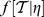 and 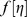 are relevant, all other terms are constant.

For the analysis of the simulated trees, we assumed that the epidemiological parameters did not change at any time during the time span encompassed by the phylogenetic tree; meaning we assumed simple exponential growth of the epidemic. We explored performance of the MCMC implementation of the birth-death skyline serial model with 1 interval and the coalescent model with exponential growth rate as implemented in BEAST v2.0 (birth-death skyline model in add-on BDSKY [25]). For the analyses explicitly mentioning use of the birth-death skyline model, we used 10 intervals for the sampling probability.

The expression for 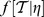 under the exponential growth coalescent and the birth-death model have been derived previously [12], [23], [24]. As the goal of our paper is to identify the impact of using either of these formulations for inferring the epidemiological parameters, we will state the mathematical expressions here.

For stating 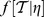 under the coalescent, we need the following definitions. We measure time going backwards from present. Present time is defined to be 0. Let 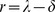 be the growth rate, 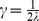 be the duration of one generation in calendar units (equal to the inter-transmission interval length) [14], and 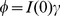, which leads to 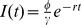. Thus, 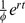 is the instantaneous rate of any pair of lineages merging. Let 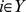 be an internal node at time 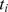 of the tree (if we have 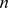 leaves in the tree, the number of internal nodes is 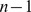), corresponding to, going back in time, a coalescent event. Let 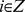 be a tip at time 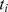 (total of 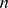) and 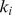 be the number of lineages co-existing in the interval between time 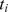 and 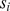, where 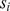 is the time of the node (internal node or tip) occurring directly after (i.e. more recent) than node 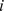 (if 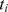 = 0, we set 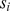 = 0). Then, from [12], corrected by [44],
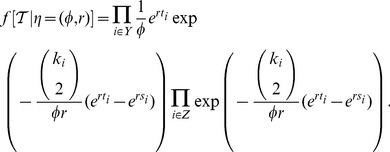
(2)

We assume throughout that the effective population size 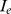 equals the infected population size, i.e. 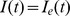.

For calculating 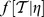 under the birth-death model with serial sampling, we need the following definitions: 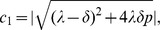

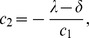

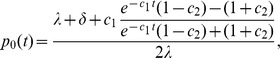

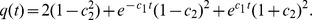


Note that 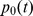 is the probability that an individual at time 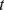 in the past will have no sampled descendants [23].

The probability 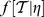 under the birth-death process, conditioned on the epidemic circulating for time 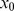 before the present (i.e. the first lineage appeared at time 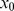), and conditioned on sampling at least one infected individual, is [23], [24], 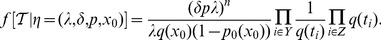
(3)

For sampling all tips at one point in time, we again use the birth-death skyline model in add-on BDSKY [25], now with sampling probability through time being 0 and present-day sampling probability being 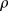.

### MCMC procedure

To sample from the posterior distribution of the parameters of interest, we apply the Markov chain Monte Carlo (MCMC) computational procedure, which explores the posterior parameter surface by taking samples from their combined posterior distribution. We run the MCMC chain in BEAST v2.0 [30] for 2,000,000 steps and sample every 1,000th step to obtain an effective sampling size (ESS) for each parameter of 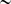 800, or higher. For this purpose, we examined the log output file from BEAST in Tracer [47]. We remove the first 200,000 steps (10%) as burn-in. For each parameter on each simulated tree, we plot the 95% highest posterior density (HPD) interval, meaning the shortest interval containing 95% of the posterior samples.

We picked uniform priors for all parameters. Priors for the two parameters 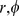 in the coalescent model with deterministic exponential population growth (available in BEAST v2.0) were chosen as:

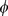
: the prior was set to a uniform distribution over interval [0,10000000], with starting value of 0.3 (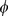 is called ePopSize in BEAST)
2. growth rate 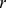: the prior was set to a uniform distribution over interval [-150000,150000], with starting value of 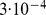.

In the birth-death model, we put priors on 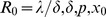. We used the birth-death skyline serial model, setting the interval number to 1, available in the BEAST v2.0 add-on BDSKY [25]:

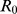
: prior was set to a uniform distribution over interval [0,1000000], with starting value of 2.0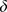: prior was set to a uniform distribution over interval [0,200000], with starting value of 1.0
3. sampling probability 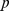: we set this quantity to the true value (under which the birth-death trees were simulated) to enable recovery of 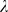 and 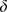; alternatively, we fix it to false 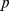, or we set a uniform prior over [0,1]
4. time between the origin 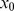 of the tree and the earliest branching event in tree (called orig_root in BEAST): prior was set to a log normal distribution with parameters M = 1 and S = 1.25 and upper limit of 1000, with starting value of 1.0.

For the birth-death skyline analyses we set the interval number for sampling probability 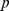 to 10. For sampling all the tips at one point in time, 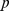 is fixed to 0 and 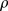 is set to the value used for tree simulation. For the remaining parameters, the same priors as above were used.

## Supporting Information

Figure S1**Comparison of the birth-death model and the coalescent model in estimating epidemic growth rate at **
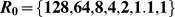
**.** For each plot, 100 trees simulated under the constant rate birth-death (BD) model with incomplete sampling (A, C, E,…) or coalescent (CE) model with exponential growth of the infected population (B, D, F,…) using various parameter combinations (see the header of each subfigure) were analyzed. See the legend of Figure 1 for detailed description.(TIF)

Figure S2**Tracking of cumulative infecteds, infecteds and sampled individuals over time in the birth-death tree simulations: **
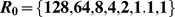
**.** For each selected 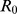 (see title of each subfigure) we simulated 100 trees under the birth-death model and counted the number of cumulative infected, infected and cumulative sampled and LTT sampled individuals at each time step. The x-axis represents the time, going backwards from present 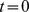, and y-axis the counts. Each line represents history of one tree over time. The left column (denoted “CE”) shows the trajectories colored on the basis of whether, within the 95% HPD interval, the coalescent was able to correctly recover (grey lines) or not (red lined for over- and orange lines for under-estimated) the true growth rate parameter from the corresponding trees. The right column (denoted “BD”) shows the same for birth-death process applied to the same trees, with purple-colored lines indicating trajectories of trees for which the growth rate was overestimated, and blue lines marking trees whose growth rate was underestimated. The abbreviations in the legend stand for the following; BD - birth-death skyline serial model (with 1 interval), CE - coalescent with deterministic exponential growth of infected population, LTT - lineages-through-time.(TIF)

Figure S3**Influence of fixing the age on the growth rate parameter estimation.** For setting 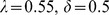 and 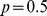 (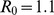), we modified the sampling scheme as not to stop simulation when 100 tips are sampled but rather when the tree age reaches certain value: 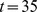 for subfigures A, B and 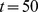 for subfigures C, D. Again, both birth-death model trees (A, C) and the coalescent trees (B, D) were simulated and analysed.(TIF)

Figure S4**Influence of increasing the sample size on the growth rate parameter estimation.** For setting 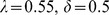 and 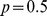 (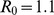), we modified the sampling scheme. We do not stop simulation when 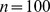 tips are sampled, but rather when more tips are sampled: 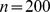 for subfigures A, B, 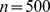 for subfigures C, D, or 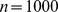 for subfigures E, F.(TIF)

Figure S5**Influence of branch length extension in various parts of the tree on the growth rate parameter estimation at **
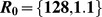
**.** We display a summary in form of the median values of the start and the end of the 95% HPD intervals, and the median of the medians of the posterior estimates for all 100 trees per each setting: 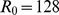 for subfigures A, B and 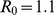 for subfigures C, D. See the legend of Figure 2 for detailed description.(TIF)

Figure S6**Influence of sampling scheme on the growth rate parameter estimation at **
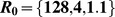
**.** We display a summary in form of the median values of the start and the end of the 95% HPD intervals, and the median of the medians of the posterior estimates for all 100 trees per setting: 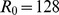 for subfigures A, B and G, H, 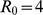 for subfigures C, D and I, J, and 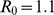 for subfigures E, F and K, L. Subfigures A-F represent sampling schemes with 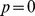 alternating with 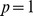 and subfigures G-L represent sampling schemes with 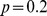 alternating with 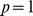. Note that for some settings, e.g. 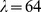, 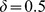, 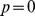 from 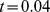 to 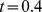, we could not simulate trees within reasonable time limit (7 days) and thus could not assess the effects of such sampling scheme alterations on parameter estimation. See the legend of Figure 3 for detailed description.(TIF)

Figure S7**Interchangeability of sampling probability in estimation of the growth rate parameter by the birth-death model.** We analyzed the 100 trees simulated with the birth-death (A, C, E,…) and the coalescent (B, D, F,…) at 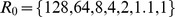 and 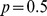 under the birth-death model assuming either the true 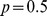 (purple bars) or untrue 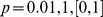 (green bars). Within each figure the trees are ordered (x-axis) by the median value of growth rate parameter estimated by the coalescent from birth-death trees. The graphs display the 95% HPD and the median (corresponding color dot within each HPD interval) of the growth rate 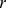 parameter. The value of the growth rate 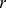 parameter under which the trees were simulated, is displayed as black horizontal bar.(TIF)

Figure S8**Interchangeability of sampling probability in estimation of **
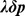
** parameter by the birth-death model.** Simulations and analyses are the same as in Figure S7, however, this time displaying 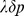. Shown are the results of analyses of the birth-death trees (A, C, E,…) and the coalescent trees (B, D, F,…) simulated under various settings: 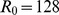 for subfigures A-F, 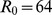 for subfigures G-L, 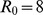 for subfigures M-R, 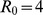 for subfigures S-X, 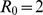 for subfigures Y-DD, 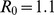 for subfigures EE-JJ, and 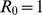 for subfigures KK-PP. Within each figure the trees are ordered (x-axis) by the median value of growth rate parameter estimated by the coalescent from birth-death trees. The value of the 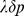 parameter under which the trees were simulated, is displayed as black horizontal bar. See Figure S7 for detailed description.(TIF)

Figure S9**Interchangeability of sampling probability in estimation of **
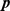
** parameter by the birth-death model.** Simulations and analyses are the same as in Figure S7, however, this time displaying 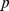. Shown are the results of analyses of the birth-death trees (A, C, E,…) and the coalescent trees (B, D, F,…) simulated under various settings: 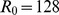 for subfigures A-F, 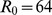 for subfigures G-L, 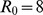 for subfigures M-R, 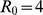 for subfigures S-X, 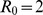 for subfigures Y-DD, 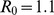 for subfigures EE-JJ, and 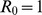 for subfigures KK-PP. Within each figure the trees are ordered (x-axis) by the median value of growth rate parameter estimated by the coalescent from birth-death trees. The value of the 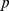 parameter under which the trees were simulated, is displayed as black horizontal bar. See Figure S7 for detailed description.(TIF)

Figure S10**Interchangeability of sampling probability in estimation of **
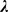
** parameter by the birth-death model.** Simulations and analyses are the same as in Figure S7, however, this time displaying 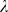. Shown are the results of analyses of the birth-death trees (A, C, E,…) and the coalescent trees (B, D, F,…) simulated under various settings: 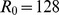 for subfigures A-F, 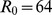 for subfigures G-L, 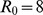 for subfigures M-R, 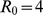 for subfigures S-X, 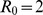 for subfigures Y-DD, 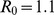 for subfigures EE-JJ, and 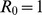 for subfigures KK-PP. Within each figure the trees are ordered (x-axis) by the median value of growth rate parameter estimated by the coalescent from birth-death trees. The value of the 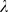 parameter under which the trees were simulated, is displayed as black horizontal bar. See Figure S7 for detailed description.(TIF)

Figure S11**Interchangeability of sampling probability in estimation of **
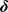
** parameter by the birth-death model.** Simulations and analyses are the same as in Figure S7, however, this time displaying 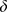. Shown are the results of analyses of the birth-death trees (A, C, E,…) and the coalescent trees (B, D, F,…) simulated under various settings: 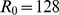 for subfigures A-F, 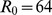 for subfigures G-L, 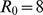 for subfigures M-R, 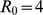 for subfigures S-X, 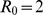 for subfigures Y-DD, 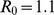 for subfigures EE-JJ, and 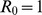 for subfigures KK-PP. Within each figure the trees are ordered (x-axis) by the median value of growth rate parameter estimated by the coalescent from birth-death trees. The value of the 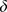 parameter under which the trees were simulated, is displayed as black horizontal bar. See Figure S7 for detailed description.(TIF)

Figure S12**Interchangeability of sampling probability in estimation of **
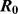
** parameter by the birth-death model.** Simulations and analyses are the same as in Figure S7, however, this time displaying 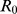. Shown are the results of analyses of the birth-death trees (A, C, E,…) and the coalescent trees (B, D, F,…) simulated under various settings: 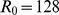 for subfigures A-F, 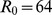 for subfigures G-L, 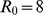 for subfigures M-R, 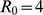 for subfigures S-X, 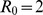 for subfigures Y-DD, 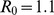 for subfigures EE-JJ, and 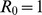 for subfigures KK-PP. Within each figure the trees are ordered (x-axis) by the median value of growth rate parameter estimated by the coalescent from birth-death trees. The value of the 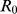 parameter under which the trees were simulated, is displayed as black horizontal bar. See Figure S7 for detailed description.(TIF)

Figure S13**Fixing **
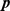
** or **
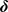
** in parameter estimation by the birth-death model.** Trees simulated with 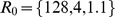 under the constant rate birth-death model - left-hand column (A, C, E,…) or under the coalescent model assuming deterministic exponentially growing population - right-hand column (B, D, F,…), were analyzed under the birth-death model assuming either fixed true 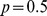 or fixed true 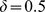. Within each figure the trees are ordered (x-axis) by the median value of growth rate parameter estimated by the coalescent from birth-death trees. The graphs display the 95% HPD and the median (corresponding color dot within each HPD interval) of each parameter: growth rate 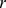, 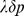, 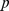, 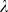, 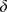, 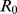, in turn. The value of each parameter under which the trees were simulated, is displayed as black horizontal bar.(TIF)

Figure S14**Effect of different information used in the **
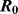
** parameter inference at **
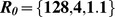
**.** From trees simulated under the setting 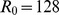 (subfigures A and B), 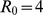 (subfigures C and D), and 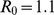 (subfigures E and F) and constant sampling 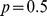, we estimated 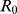 using the four methods as described in the legend of Figure 5.(TIF)

Figure S15**Comparison of the birth-death model and the coalescent model in estimating growth rate parameter from trees with tips sampled at one point in time at **
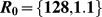
**.** For each plot, 100 trees simulated under the constant rate birth-death (BD) model at 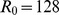 (A, C, E) or 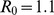 (B, D, F) using various sampling probabilities 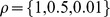 of each tip at one time point were analyzed. See the legend of Figure 6 for detailed description.(TIF)

Figure S16**Comparison of growth rate point estimates of the birth-death model and the coalescent model from trees with tips sampled at one point in time at **
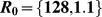
**.** For 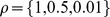, we display the ML and MAP estimates and the HPD summary for the birth-death trees with 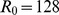 (A, C, E) and 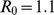 (B, D, F). See the legend of Figure 7 for detailed description.(TIF)

Figure S17**Comparison of growth rate point estimates of the birth-death model and the coalescent model for different **
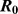
** values.** For 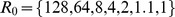 and 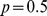, we display the ML and MAP estimates and the HPD summary for the birth-death trees (A, C, E,…) and the coalescent trees (B, D, F,…). See the legend of Figure 7 for detailed description.(TIF)

Figure S18**Comparison of growth rate point estimates of the birth-death model and the coalescent model for different sampling probabilities.** For 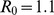 and 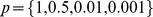, we display the ML and MAP estimates and the HPD summary for the birth-death trees (A, C, E,…) and the coalescent trees (B, D, F,…). See the legend of Figure 7 for detailed description.(TIF)

Table S1**Summary of growth rate parameter estimation statistics at various **
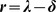
** corresponding to the same **
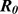
**.** For each of the 100 trees simulated under the respective model (the birth-death or the coalescent), with 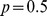 and various growth rates 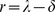 corresponding to 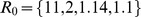, we estimated the coverage, the 95% HPD interval sizes and RMSE of 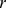 by the birth-death model and the coalescent model, and display the summary of these measures. Each value of 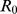 that corresponds to the same growth rate 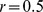 across all the 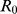 settings is marked in bold. Different simulations corresponding to the same 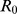 are separated by horizontal double line.(PDF)

Table S2**Summary of growth rate parameter estimation statistics in various sampling scenarios.** For each of the 100 trees simulated under the respective model (the birth-death or the coalescent), with periods of 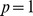 sampling probability alternating with periods of 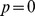 or 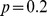, we estimated the coverage, the 95% HPD interval sizes and RMSE of 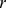 by the birth-death model (1 interval), the birth-death skyline model allowing for varying sampling proportion 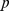 (10 equidistant intervals for 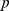) and the coalescent model, and display the summary of these measures. Different simulations corresponding to the same 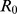 are separated by horizontal double line. 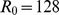 corresponds to 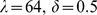, 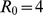 corresponds to 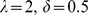 and 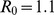 corresponds to 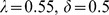.(PDF)

Table S3**Summary of growth rate parameter estimation statistics for trees with tips sampled at one point in time.** For each of the 100 trees simulated under the birth-death model, with 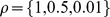 and 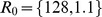, we estimated the coverage, the 95% HPD interval sizes and the RMSE of 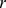 by the birth-death model and the coalescent model, and display the summary of these measures.(PDF)

Supplementary Material S1 **First part describes the derivation of waiting times until coalescent under discrete time Wright-Fisher, discrete time Moran, and continuous time Wright-Fisher and Moran population models.** Second part discusses parameter correlations under the birth-death process.
(PDF)

File S1 **An example xml file for simulation of coalescent trees, using sampling times from the birth-death process, by sampling from the coalescent tree prior in BEAST v2.0 is provided.**
(XML)
