# Supplementary figures and images for: Inference of Epidemiological Dynamics Based on Simulated Phylogenies Using Birth-Death and Coalescent Models
Source: PLoS Comput Biol. 2014 Nov 6;10(11):e1003913. doi: 10.1371/journal.pcbi.1003913 (PMC4222655; doi:10.1371/journal.pcbi.1003913)

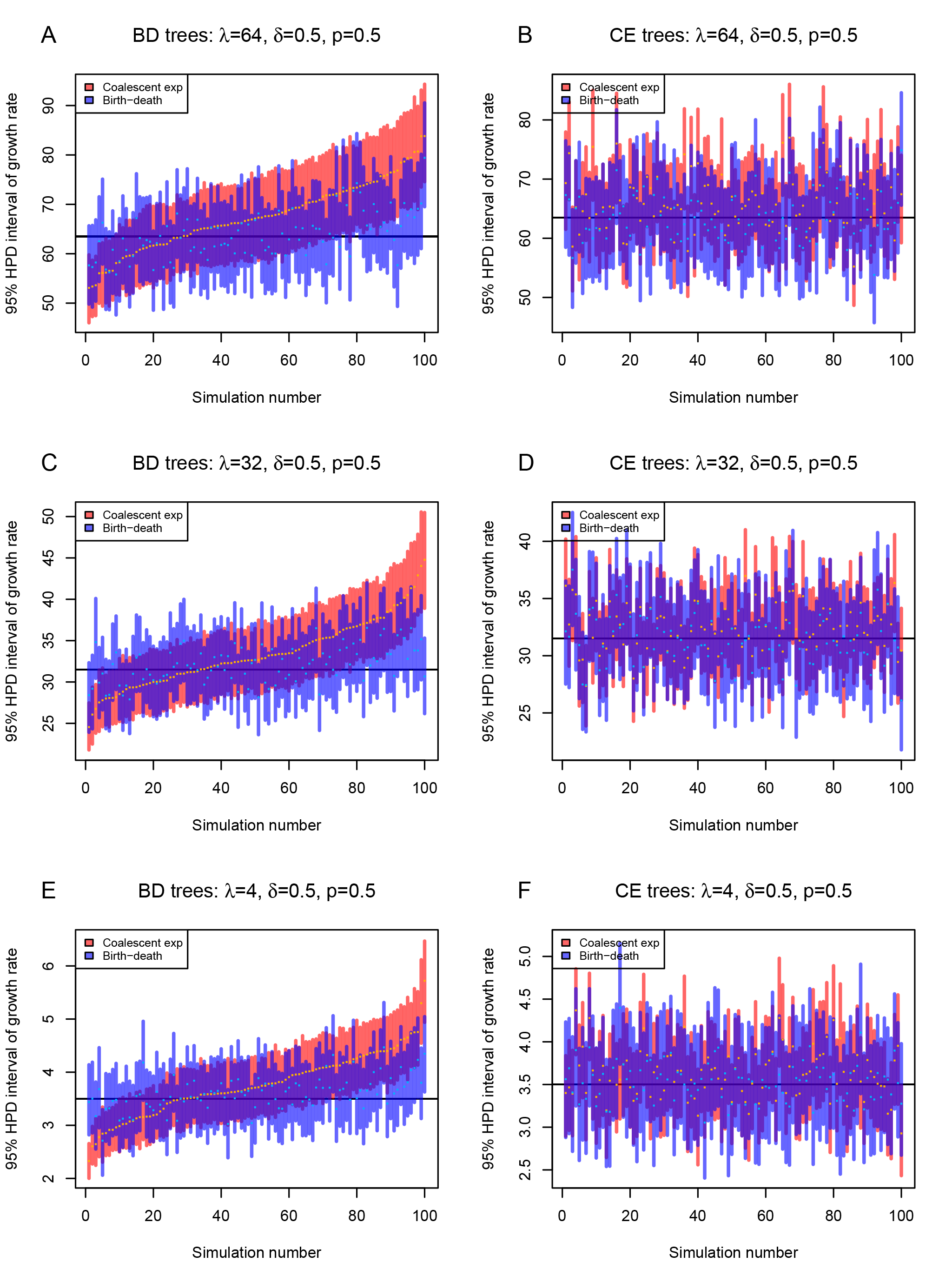

Supplement: Figure S1 — Comparison of the birth-death model and the coalescent model in estimating epidemic growth rate at . For each plot, 100 trees simulated under the constant rate birth-death (BD) model with incomplete sampling (A, C, E,…) or coalescent (CE) model with exponential growth of the infected population (B, D, F,…) using various parameter combinations (see the header of each subfigure) were analyzed. See the legend of Figure 1 for detailed description. (TIF) [file pcbi.1003913.s001.tif]

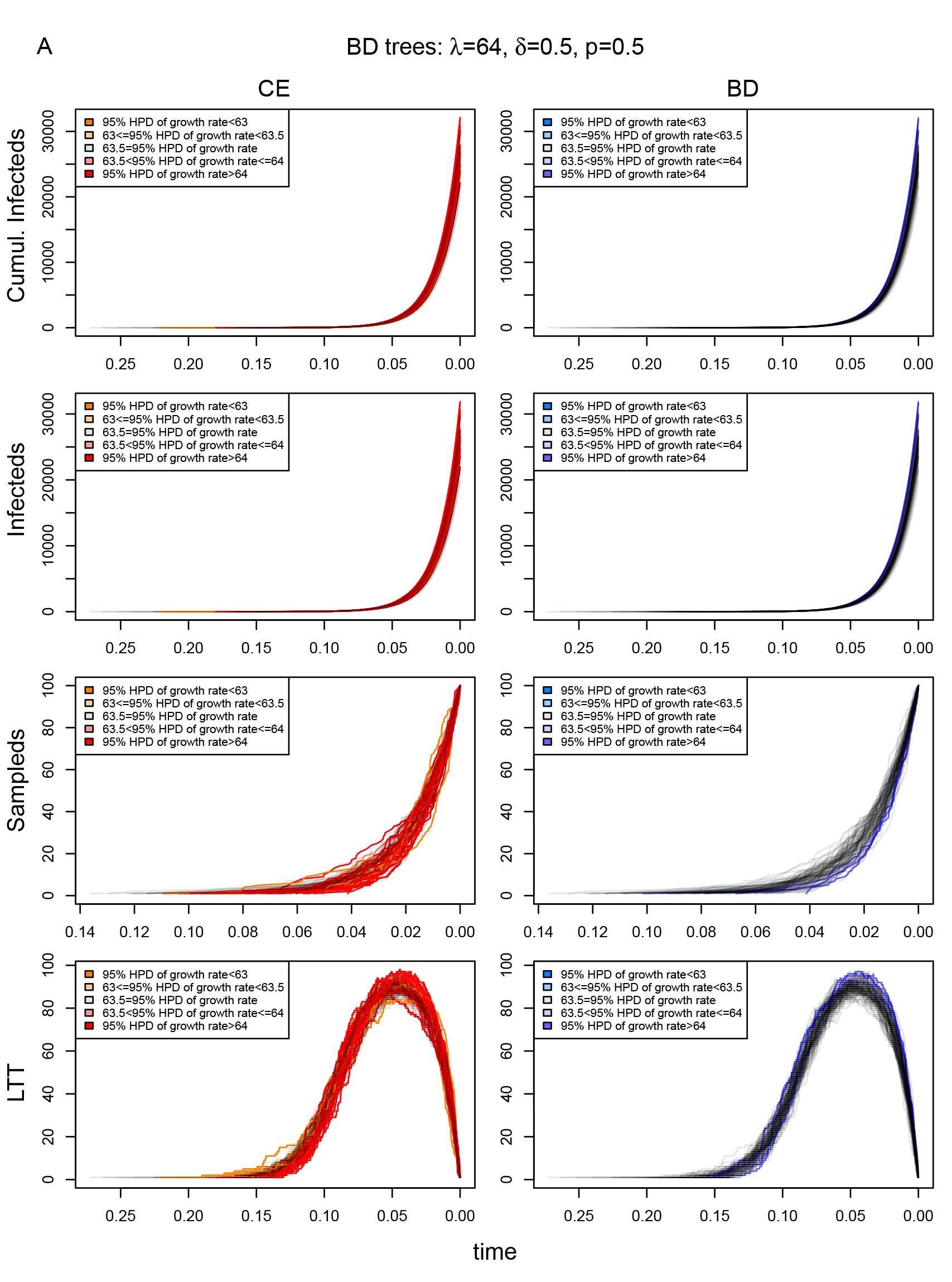

Supplement: Figure S2 — Tracking of cumulative infecteds, infecteds and sampled individuals over time in the birth-death tree simulations: . For each selected (see title of each subfigure) we simulated 100 trees under the birth-death model and counted the number of cumulative infected, infected and cumulative sampled and LTT sampled individuals at each time step. The x-axis represents the time, going backwards from present , and y-axis the counts. Each line represents history of one tree over time. The left column (denoted “CE”) shows the trajectories colored on the basis of whether, within the 95% HPD interval, the coalescent was able to correctly recover (grey lines) or not (red lined for over- and orange lines for under-estimated) the true growth rate parameter from the corresponding trees. The right column (denoted “BD”) shows the same for birth-death process applied to the same trees, with purple-colored lines indicating trajectories of trees for which the growth rate was overestimated, and blue lines marking trees whose growth rate was underestimated. The abbreviations in the legend stand for the following; BD - birth-death skyline serial model (with 1 interval), CE - coalescent with deterministic exponential growth of infected population, LTT - lineages-through-time. (TIF) [file pcbi.1003913.s002.tif]

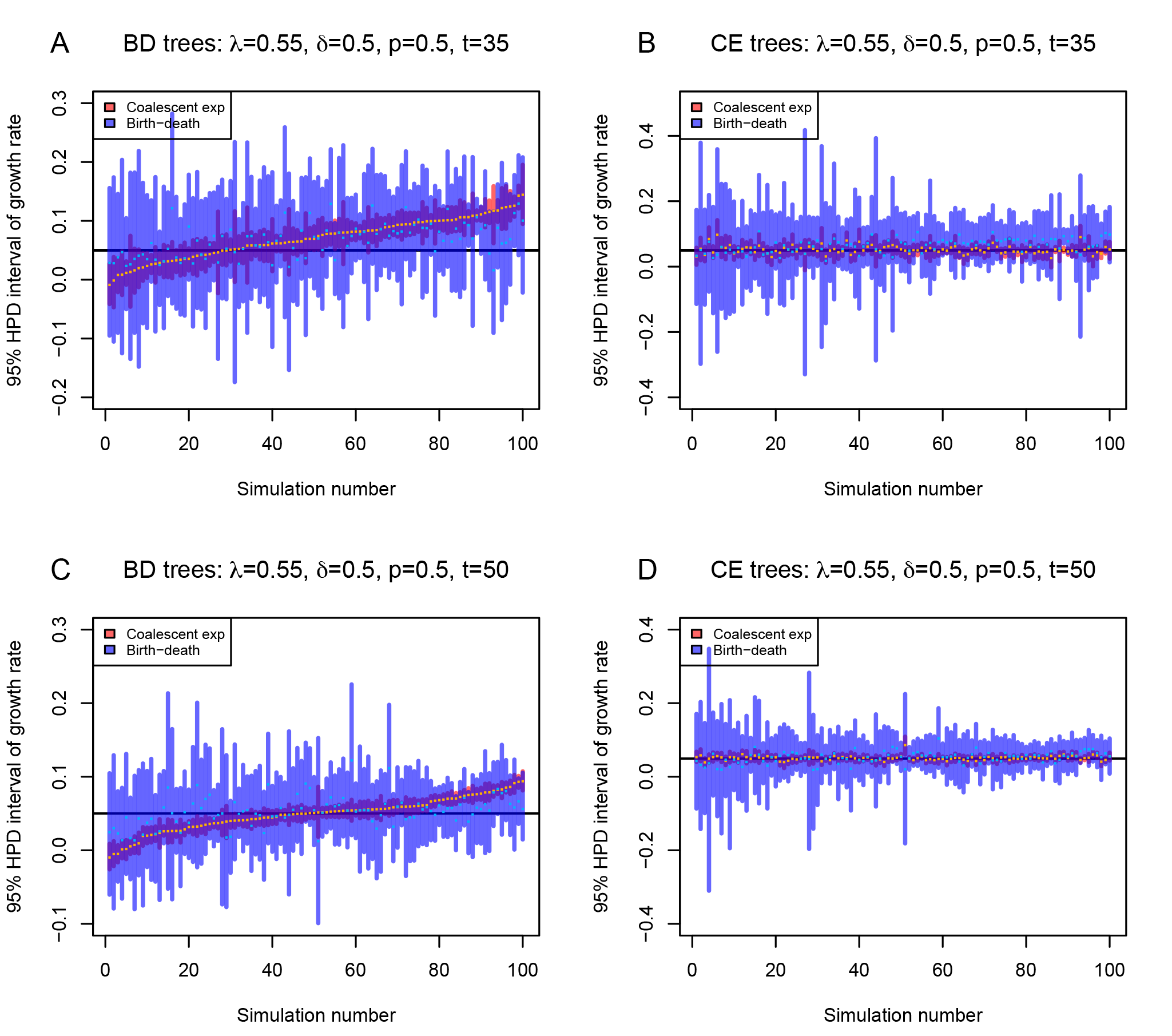

Supplement: Figure S3 — Influence of fixing the age on the growth rate parameter estimation. For setting and (), we modified the sampling scheme as not to stop simulation when 100 tips are sampled but rather when the tree age reaches certain value: for subfigures A, B and for subfigures C, D. Again, both birth-death model trees (A, C) and the coalescent trees (B, D) were simulated and analysed. (TIF) [file pcbi.1003913.s003.tif]

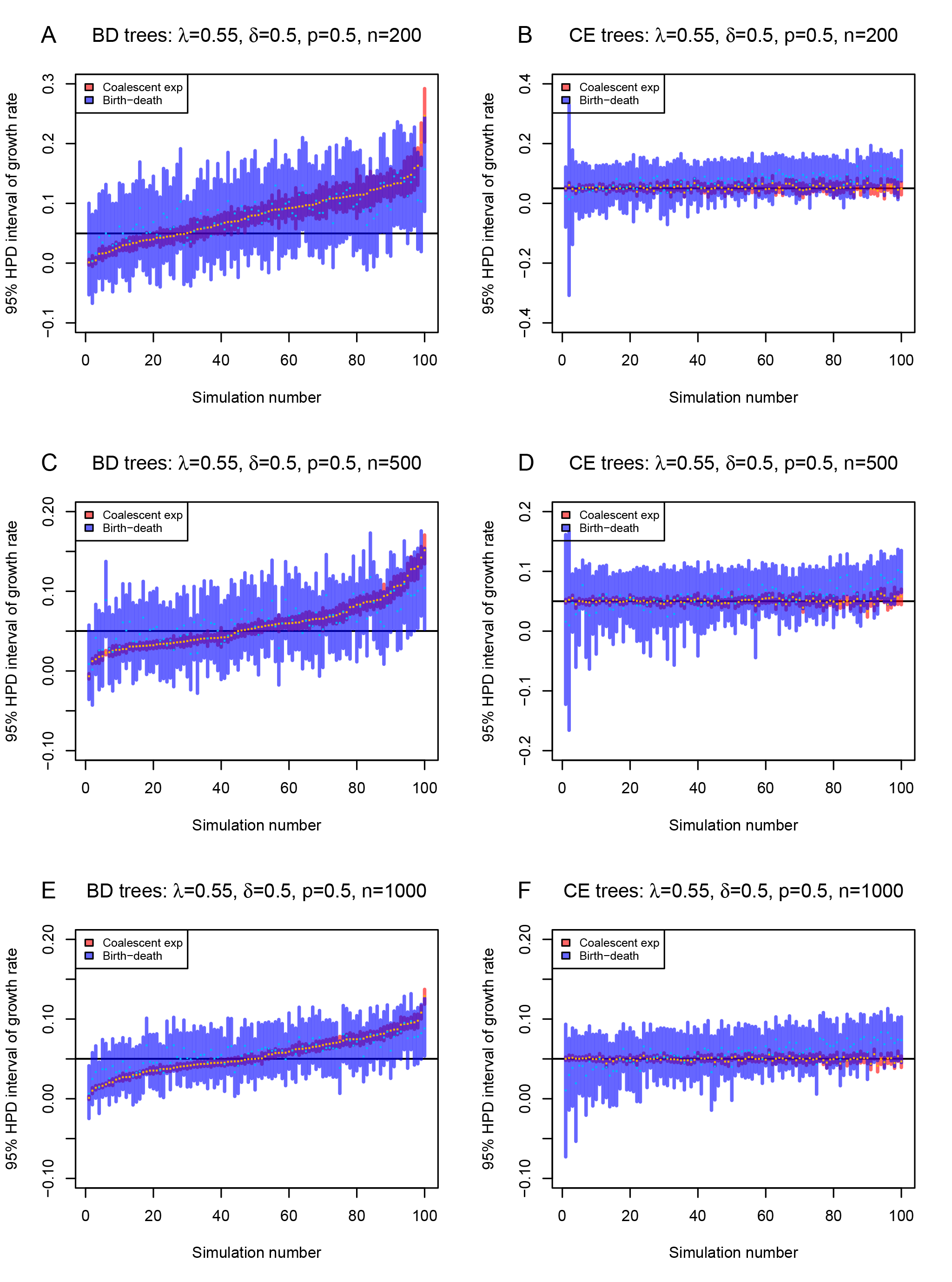

Supplement: Figure S4 — Influence of increasing the sample size on the growth rate parameter estimation. For setting and (), we modified the sampling scheme. We do not stop simulation when tips are sampled, but rather when more tips are sampled: for subfigures A, B, for subfigures C, D, or for subfigures E, F. (TIF) [file pcbi.1003913.s004.tif]

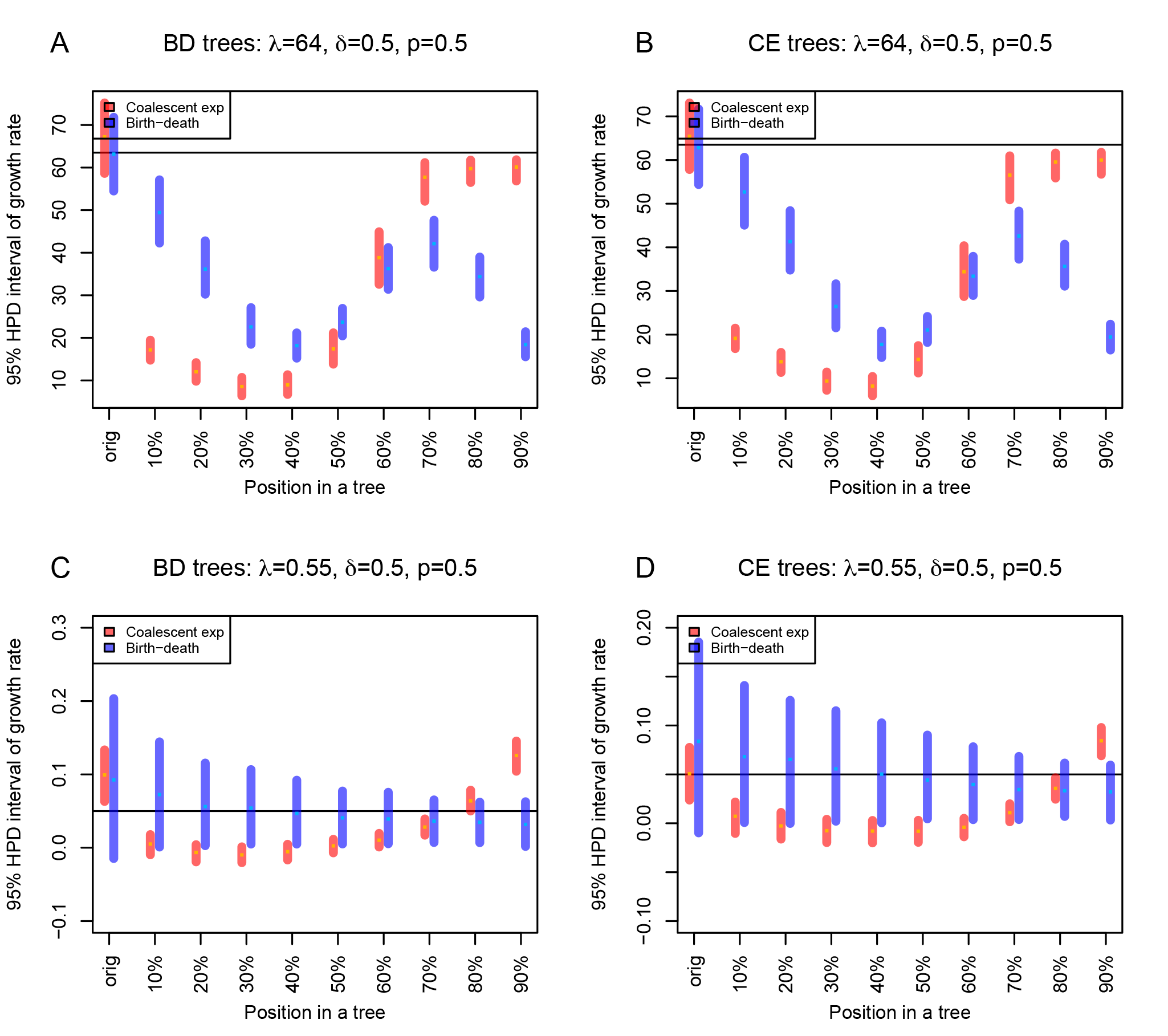

Supplement: Figure S5 — Influence of branch length extension in various parts of the tree on the growth rate parameter estimation at . We display a summary in form of the median values of the start and the end of the 95% HPD intervals, and the median of the medians of the posterior estimates for all 100 trees per each setting: for subfigures A, B and for subfigures C, D. See the legend of Figure 2 for detailed description. (TIF) [file pcbi.1003913.s005.tif]

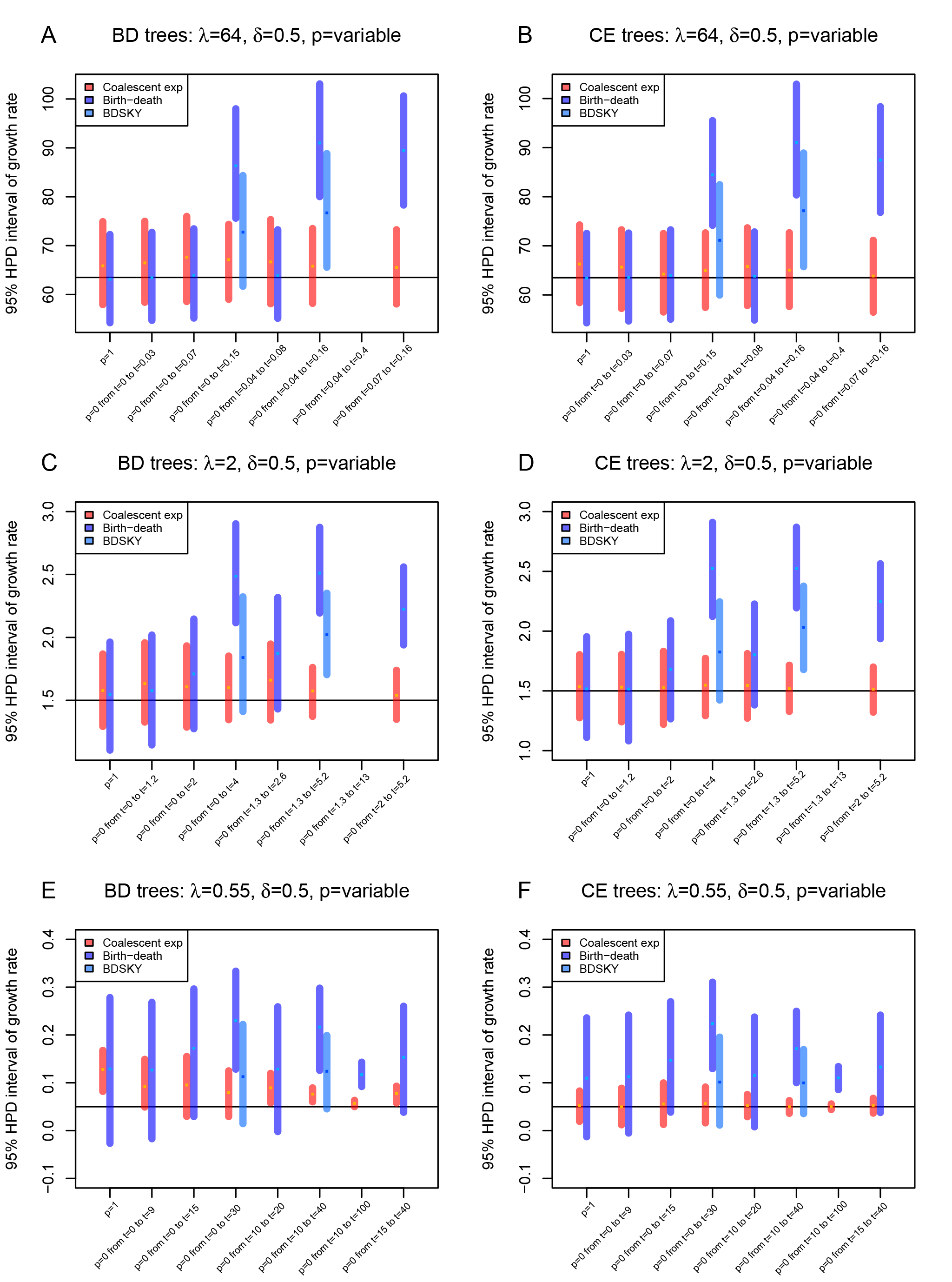

Supplement: Figure S6 — Influence of sampling scheme on the growth rate parameter estimation at . We display a summary in form of the median values of the start and the end of the 95% HPD intervals, and the median of the medians of the posterior estimates for all 100 trees per setting: for subfigures A, B and G, H, for subfigures C, D and I, J, and for subfigures E, F and K, L. Subfigures A-F represent sampling schemes with alternating with and subfigures G-L represent sampling schemes with alternating with . Note that for some settings, e.g. , , from to , we could not simulate trees within reasonable time limit (7 days) and thus could not assess the effects of such sampling scheme alterations on parameter estimation. See the legend of Figure 3 for detailed description. (TIF) [file pcbi.1003913.s006.tif]

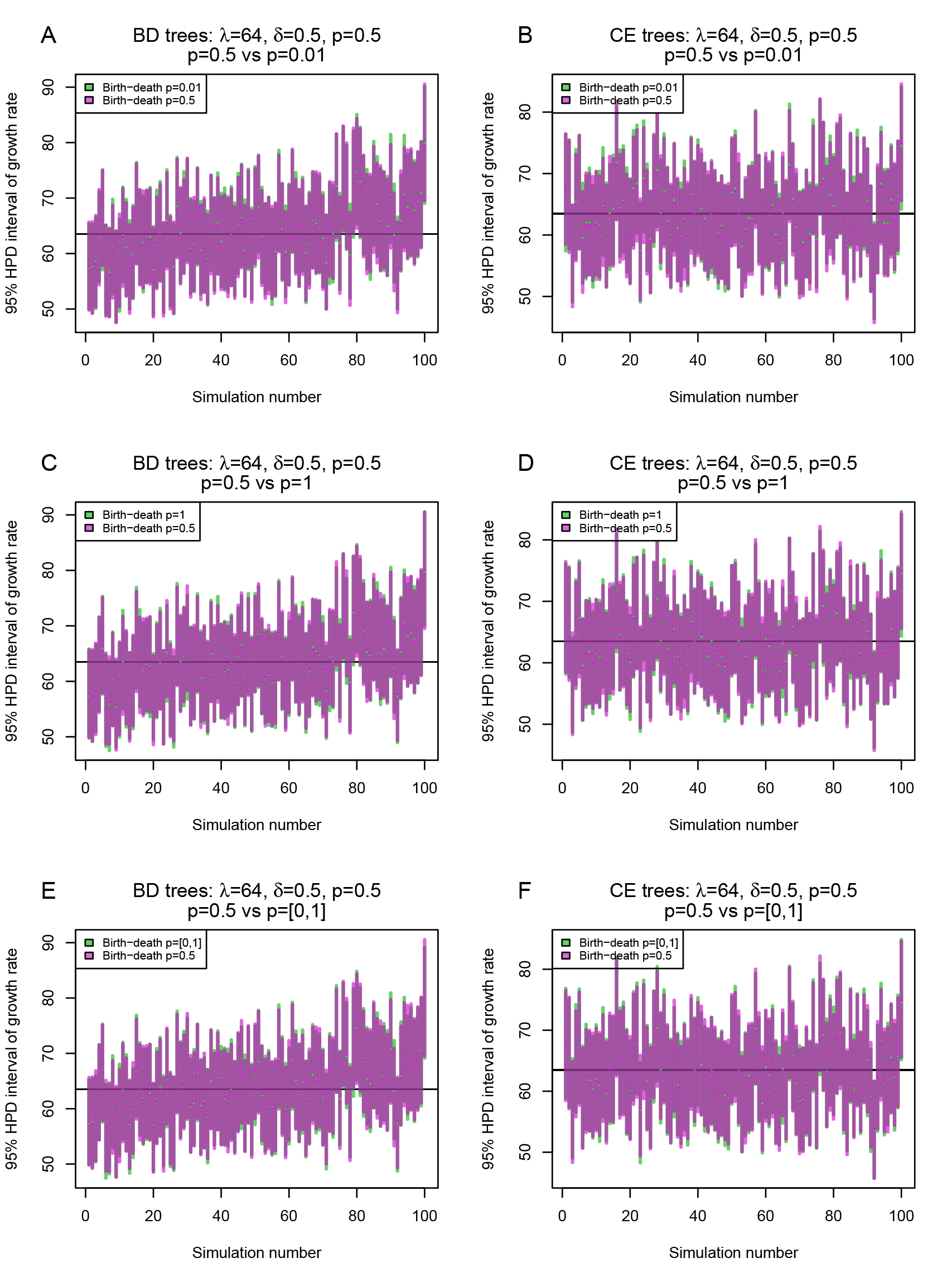

Supplement: Figure S7 — Interchangeability of sampling probability in estimation of the growth rate parameter by the birth-death model. We analyzed the 100 trees simulated with the birth-death (A, C, E,…) and the coalescent (B, D, F,…) at and under the birth-death model assuming either the true (purple bars) or untrue (green bars). Within each figure the trees are ordered (x-axis) by the median value of growth rate parameter estimated by the coalescent from birth-death trees. The graphs display the 95% HPD and the median (corresponding color dot within each HPD interval) of the growth rate parameter. The value of the growth rate parameter under which the trees were simulated, is displayed as black horizontal bar. (TIF) [file pcbi.1003913.s007.tif]

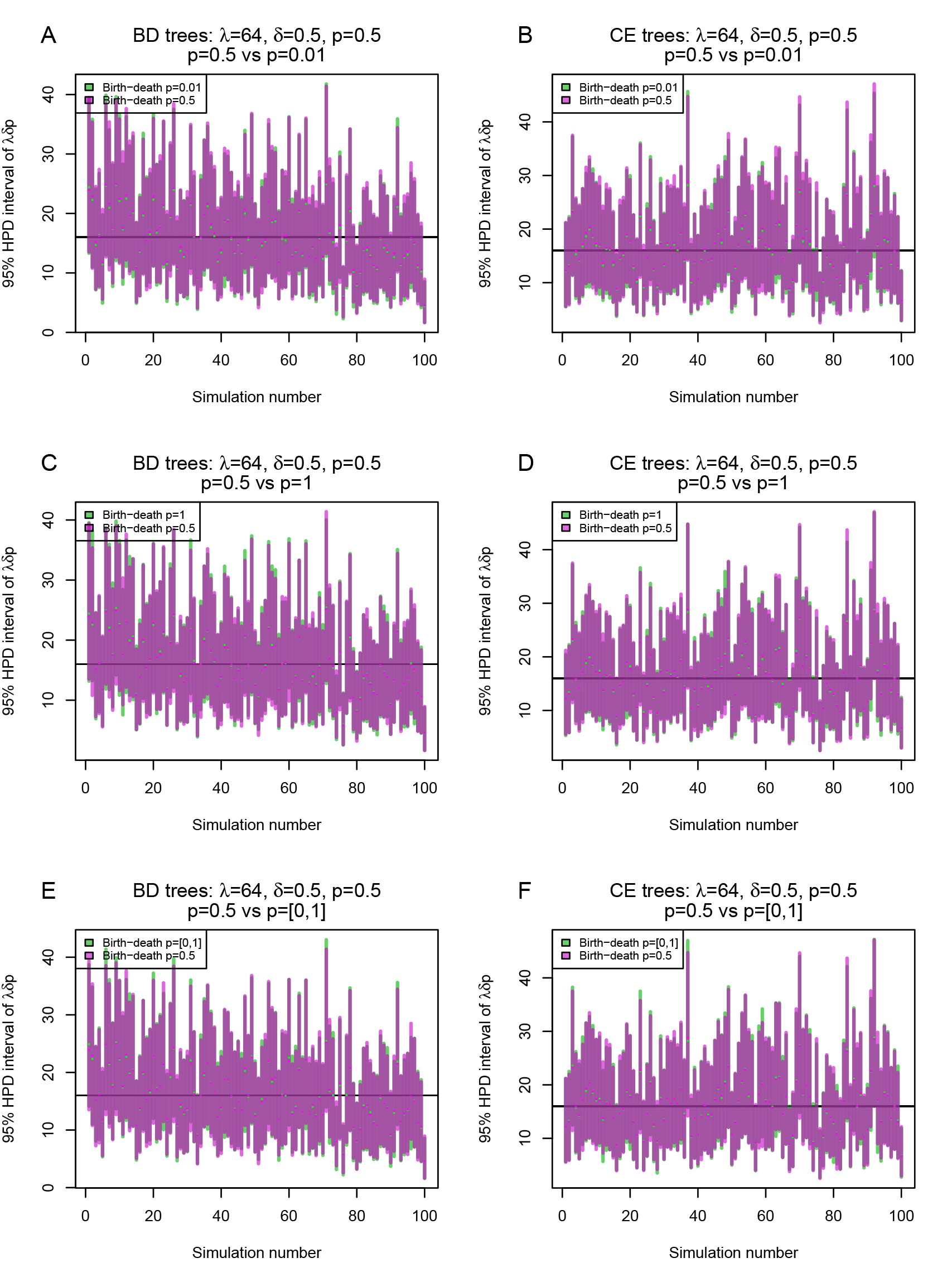

Supplement: Figure S8 — Interchangeability of sampling probability in estimation of parameter by the birth-death model. Simulations and analyses are the same as in Figure S7, however, this time displaying . Shown are the results of analyses of the birth-death trees (A, C, E,…) and the coalescent trees (B, D, F,…) simulated under various settings: for subfigures A-F, for subfigures G-L, for subfigures M-R, for subfigures S-X, for subfigures Y-DD, for subfigures EE-JJ, and for subfigures KK-PP. Within each figure the trees are ordered (x-axis) by the median value of growth rate parameter estimated by the coalescent from birth-death trees. The value of the parameter under which the trees were simulated, is displayed as black horizontal bar. See Figure S7 for detailed description. (TIF) [file pcbi.1003913.s008.tif]

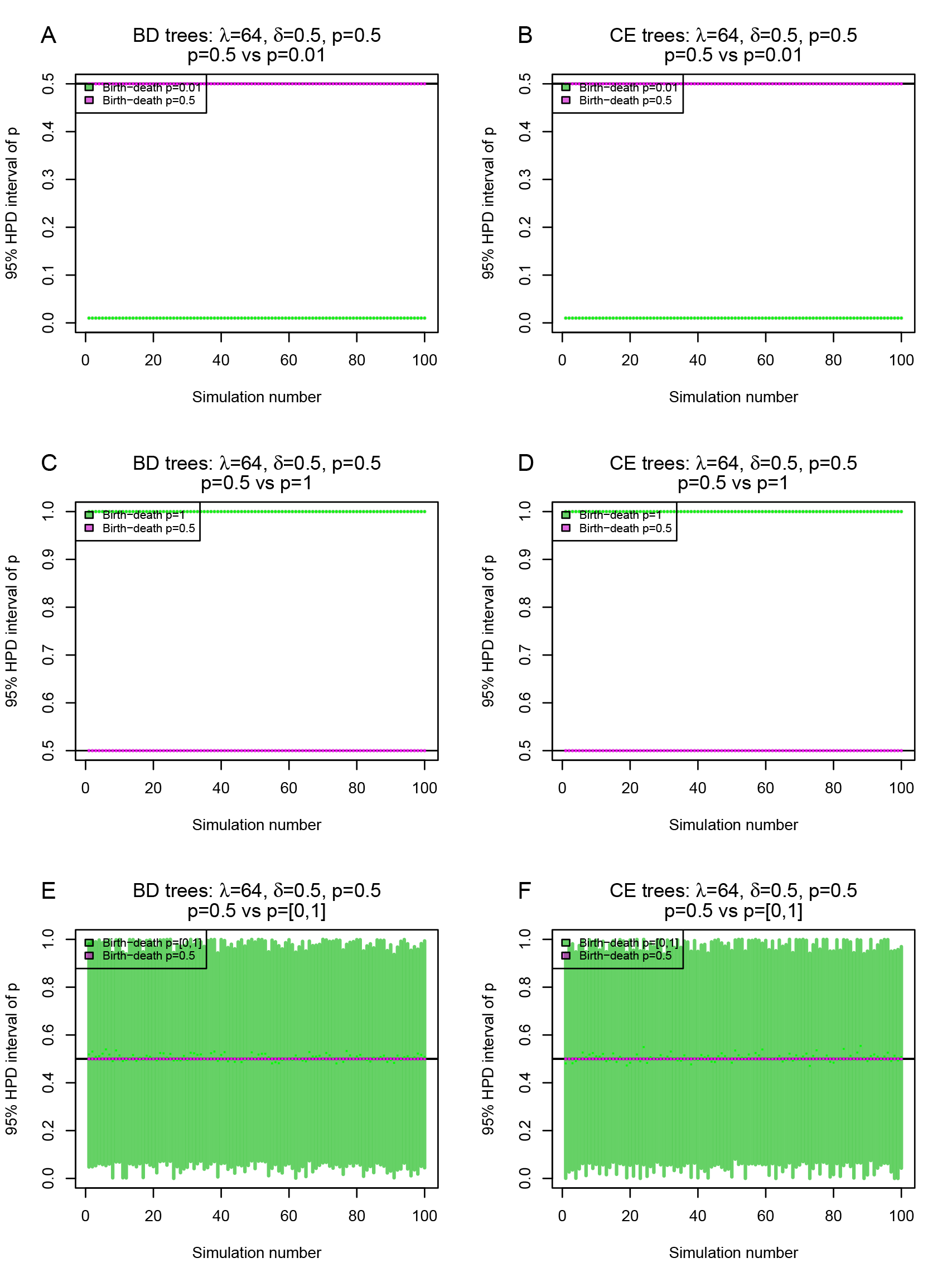

Supplement: Figure S9 — Interchangeability of sampling probability in estimation of parameter by the birth-death model. Simulations and analyses are the same as in Figure S7, however, this time displaying . Shown are the results of analyses of the birth-death trees (A, C, E,…) and the coalescent trees (B, D, F,…) simulated under various settings: for subfigures A-F, for subfigures G-L, for subfigures M-R, for subfigures S-X, for subfigures Y-DD, for subfigures EE-JJ, and for subfigures KK-PP. Within each figure the trees are ordered (x-axis) by the median value of growth rate parameter estimated by the coalescent from birth-death trees. The value of the parameter under which the trees were simulated, is displayed as black horizontal bar. See Figure S7 for detailed description. (TIF) [file pcbi.1003913.s009.tif]

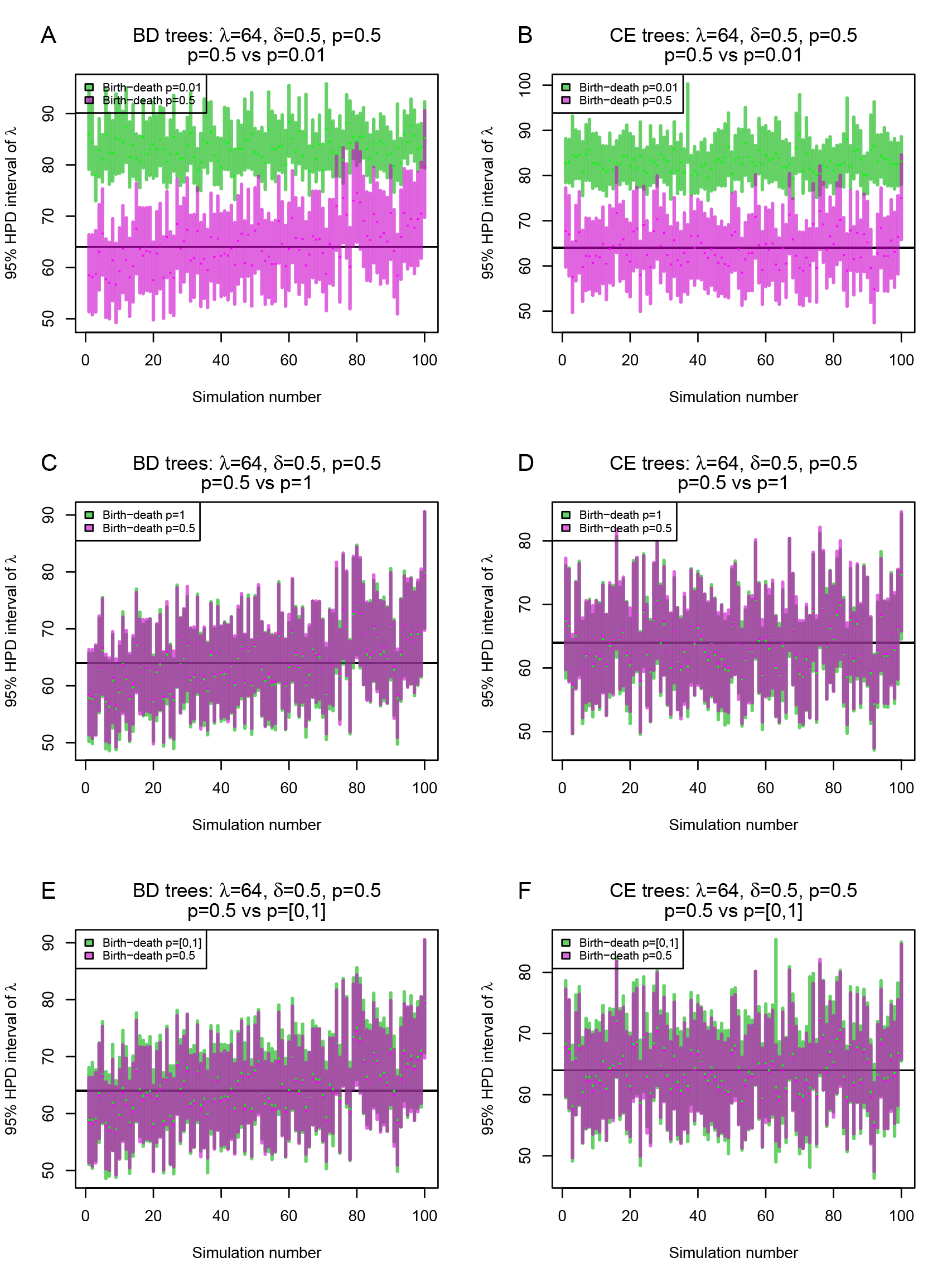

Supplement: Figure S10 — Interchangeability of sampling probability in estimation of parameter by the birth-death model. Simulations and analyses are the same as in Figure S7, however, this time displaying . Shown are the results of analyses of the birth-death trees (A, C, E,…) and the coalescent trees (B, D, F,…) simulated under various settings: for subfigures A-F, for subfigures G-L, for subfigures M-R, for subfigures S-X, for subfigures Y-DD, for subfigures EE-JJ, and for subfigures KK-PP. Within each figure the trees are ordered (x-axis) by the median value of growth rate parameter estimated by the coalescent from birth-death trees. The value of the parameter under which the trees were simulated, is displayed as black horizontal bar. See Figure S7 for detailed description. (TIF) [file pcbi.1003913.s010.tif]

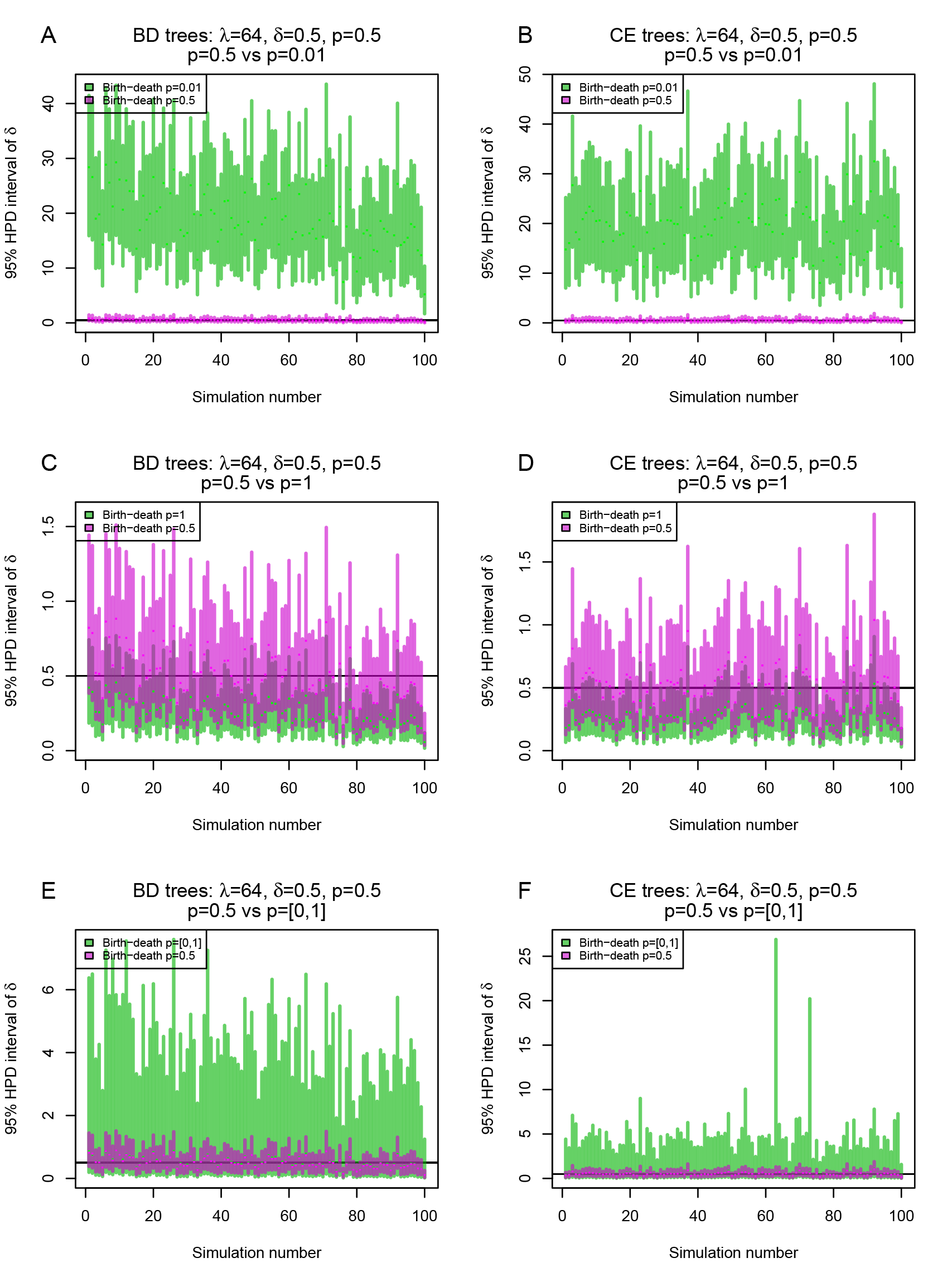

Supplement: Figure S11 — Interchangeability of sampling probability in estimation of parameter by the birth-death model. Simulations and analyses are the same as in Figure S7, however, this time displaying . Shown are the results of analyses of the birth-death trees (A, C, E,…) and the coalescent trees (B, D, F,…) simulated under various settings: for subfigures A-F, for subfigures G-L, for subfigures M-R, for subfigures S-X, for subfigures Y-DD, for subfigures EE-JJ, and for subfigures KK-PP. Within each figure the trees are ordered (x-axis) by the median value of growth rate parameter estimated by the coalescent from birth-death trees. The value of the parameter under which the trees were simulated, is displayed as black horizontal bar. See Figure S7 for detailed description. (TIF) [file pcbi.1003913.s011.tif]

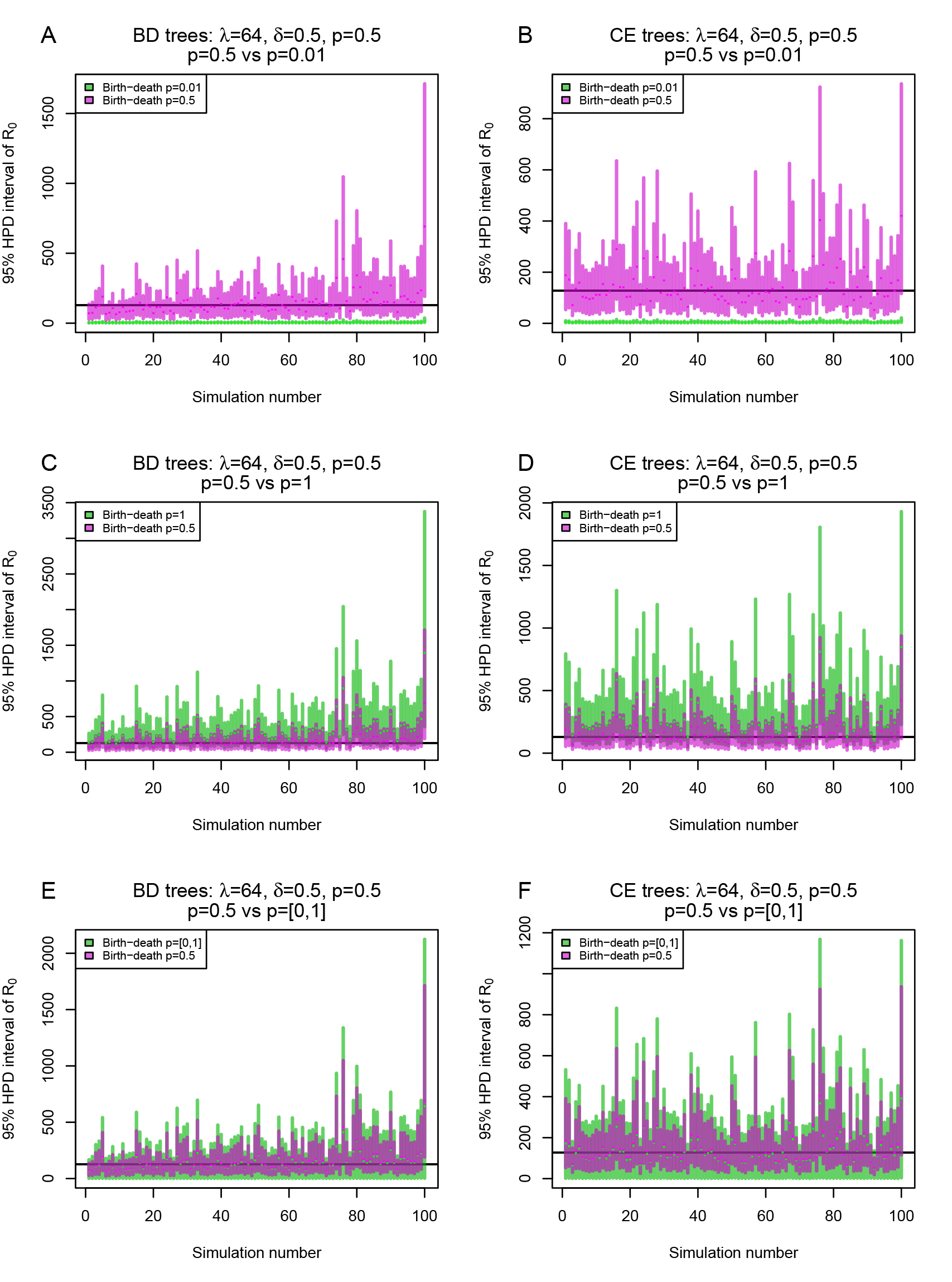

Supplement: Figure S12 — Interchangeability of sampling probability in estimation of parameter by the birth-death model. Simulations and analyses are the same as in Figure S7, however, this time displaying . Shown are the results of analyses of the birth-death trees (A, C, E,…) and the coalescent trees (B, D, F,…) simulated under various settings: for subfigures A-F, for subfigures G-L, for subfigures M-R, for subfigures S-X, for subfigures Y-DD, for subfigures EE-JJ, and for subfigures KK-PP. Within each figure the trees are ordered (x-axis) by the median value of growth rate parameter estimated by the coalescent from birth-death trees. The value of the parameter under which the trees were simulated, is displayed as black horizontal bar. See Figure S7 for detailed description. (TIF) [file pcbi.1003913.s012.tif]

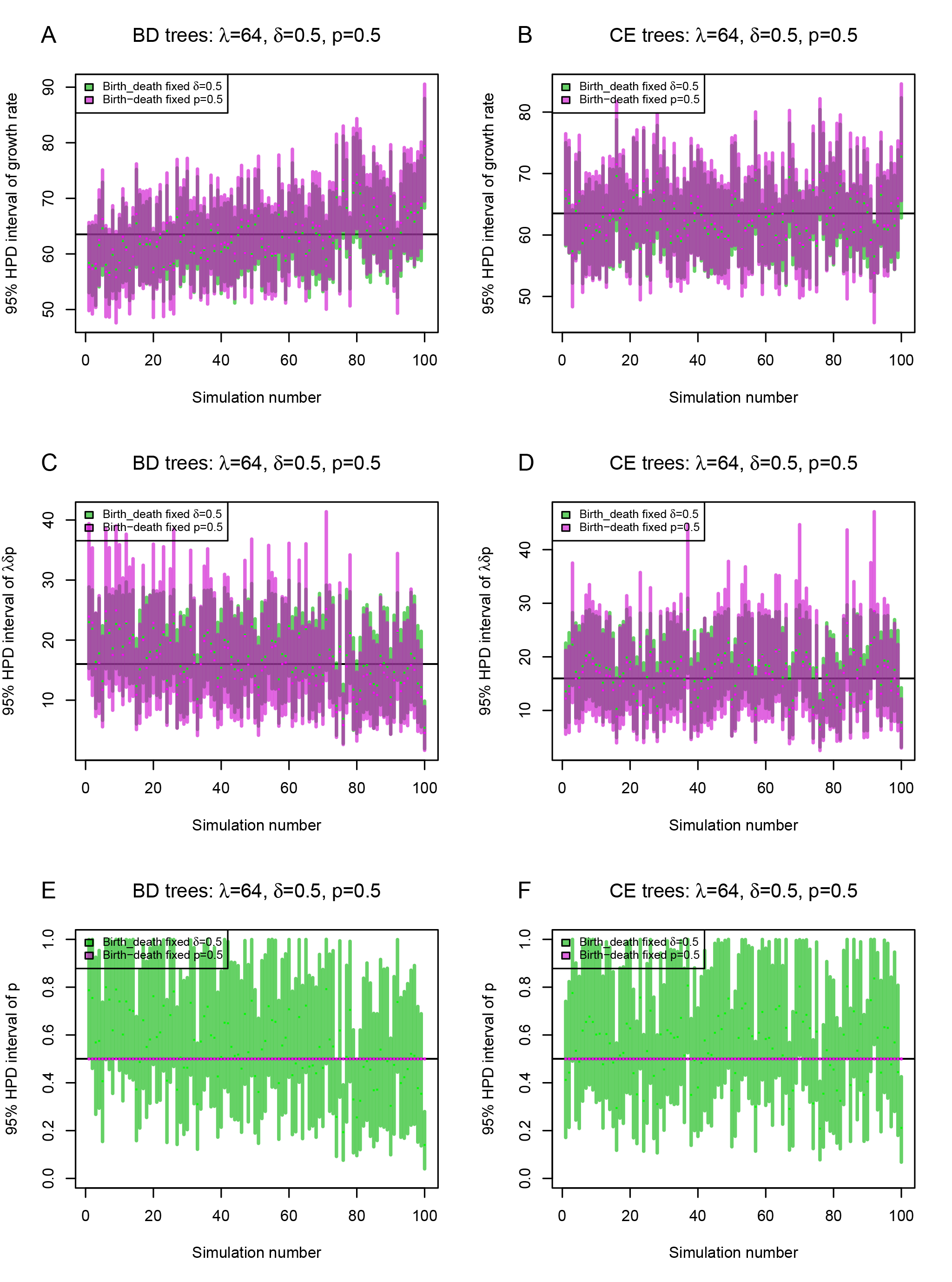

Supplement: Figure S13 — Fixing or in parameter estimation by the birth-death model. Trees simulated with under the constant rate birth-death model - left-hand column (A, C, E,…) or under the coalescent model assuming deterministic exponentially growing population - right-hand column (B, D, F,…), were analyzed under the birth-death model assuming either fixed true or fixed true . Within each figure the trees are ordered (x-axis) by the median value of growth rate parameter estimated by the coalescent from birth-death trees. The graphs display the 95% HPD and the median (corresponding color dot within each HPD interval) of each parameter: growth rate , , , , , , in turn. The value of each parameter under which the trees were simulated, is displayed as black horizontal bar. (TIF) [file pcbi.1003913.s013.tif]

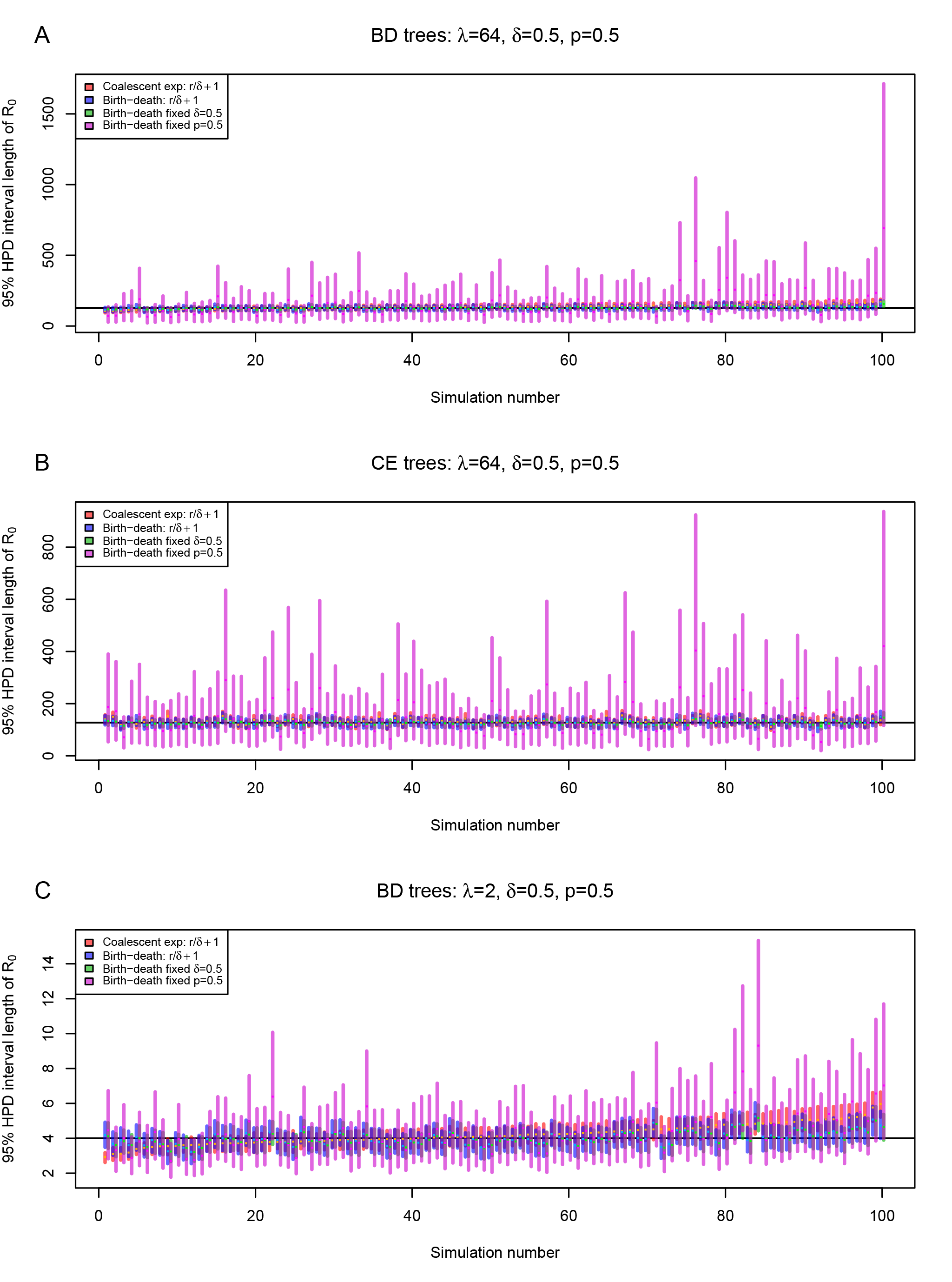

Supplement: Figure S14 — Effect of different information used in the parameter inference at . From trees simulated under the setting (subfigures A and B), (subfigures C and D), and (subfigures E and F) and constant sampling , we estimated using the four methods as described in the legend of Figure 5. (TIF) [file pcbi.1003913.s014.tif]

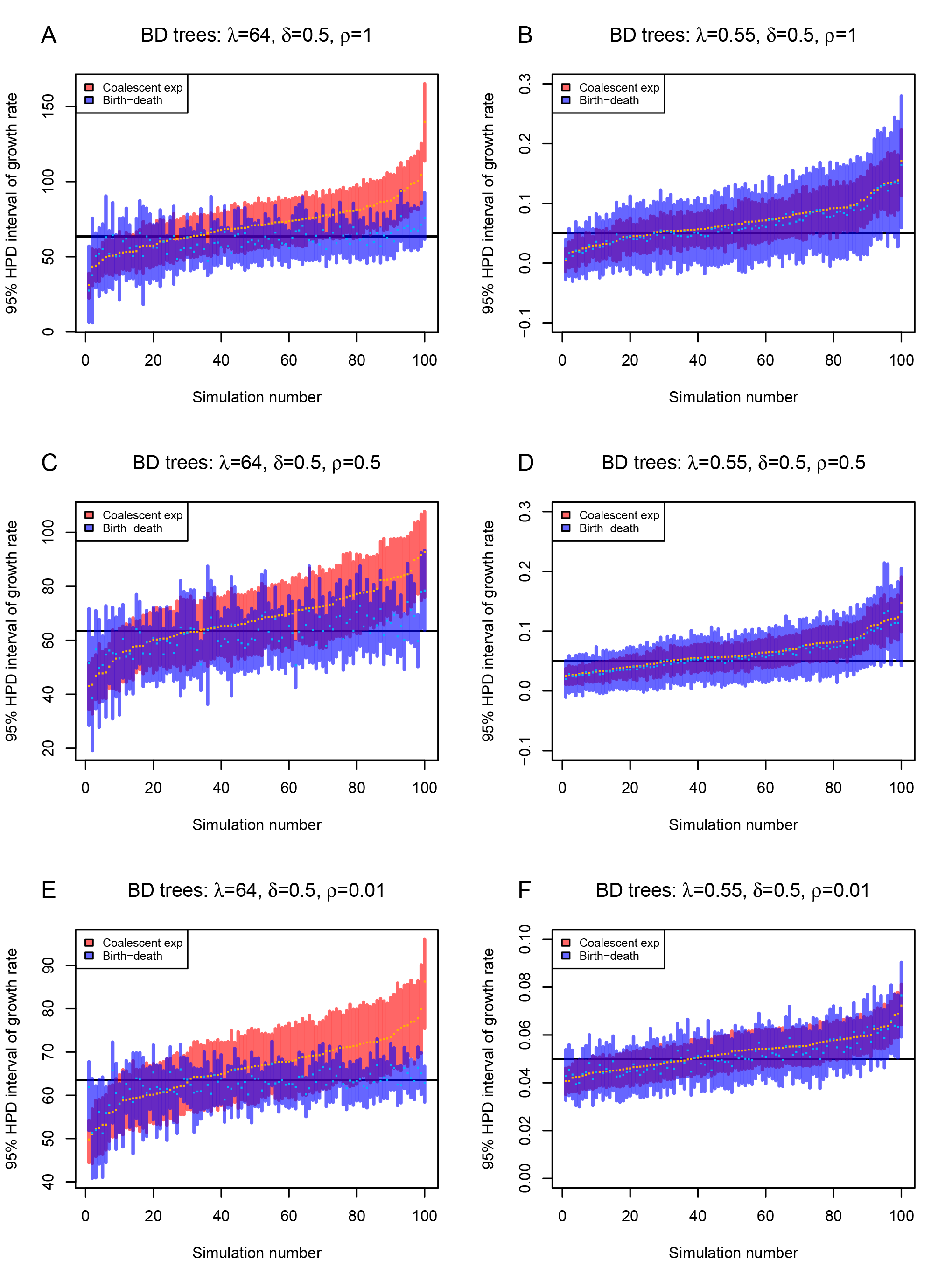

Supplement: Figure S15 — Comparison of the birth-death model and the coalescent model in estimating growth rate parameter from trees with tips sampled at one point in time at . For each plot, 100 trees simulated under the constant rate birth-death (BD) model at (A, C, E) or (B, D, F) using various sampling probabilities of each tip at one time point were analyzed. See the legend of Figure 6 for detailed description. (TIF) [file pcbi.1003913.s015.tif]

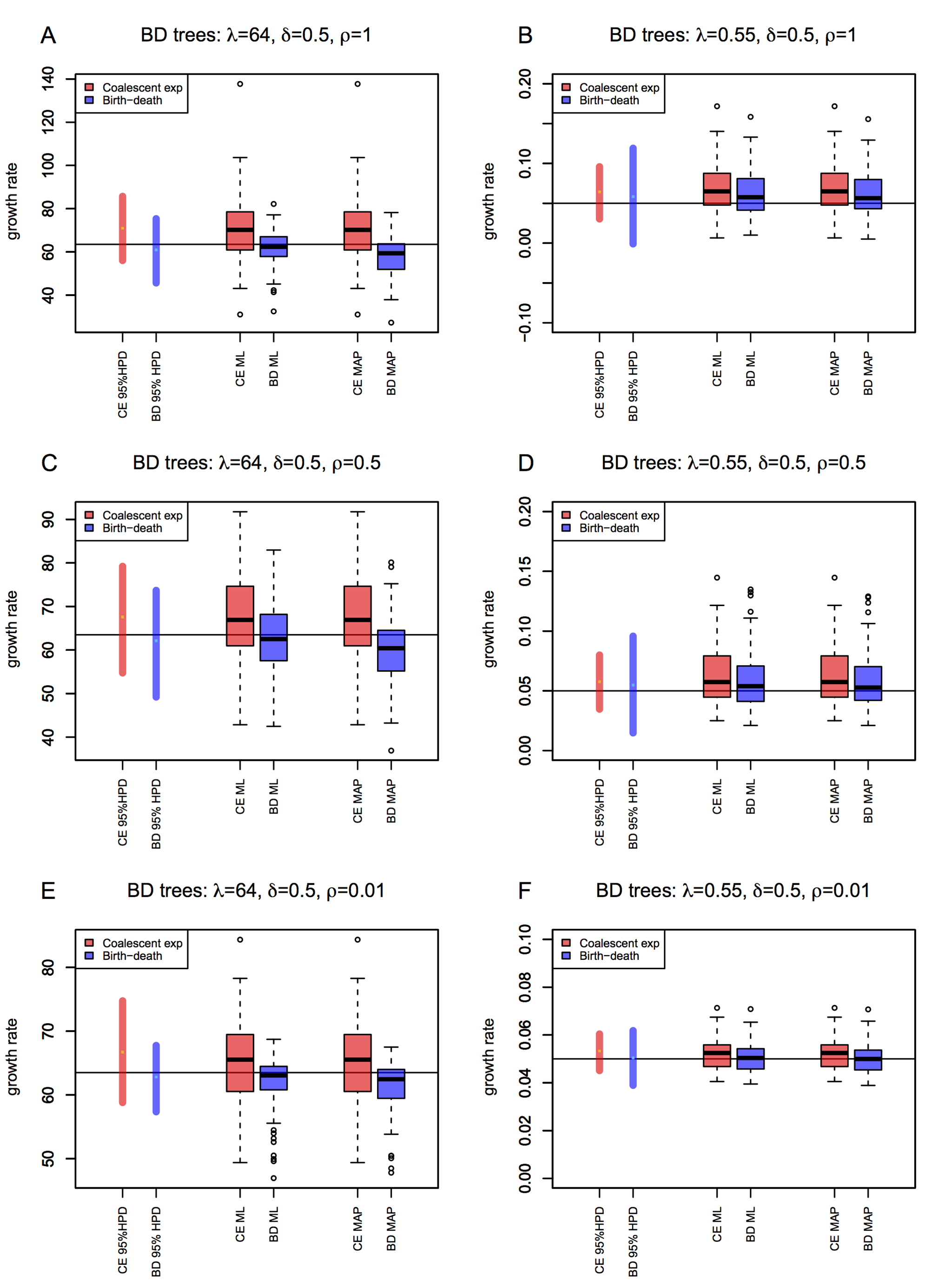

Supplement: Figure S16 — Comparison of growth rate point estimates of the birth-death model and the coalescent model from trees with tips sampled at one point in time at . For , we display the ML and MAP estimates and the HPD summary for the birth-death trees with (A, C, E) and (B, D, F). See the legend of Figure 7 for detailed description. (TIF) [file pcbi.1003913.s016.tif]

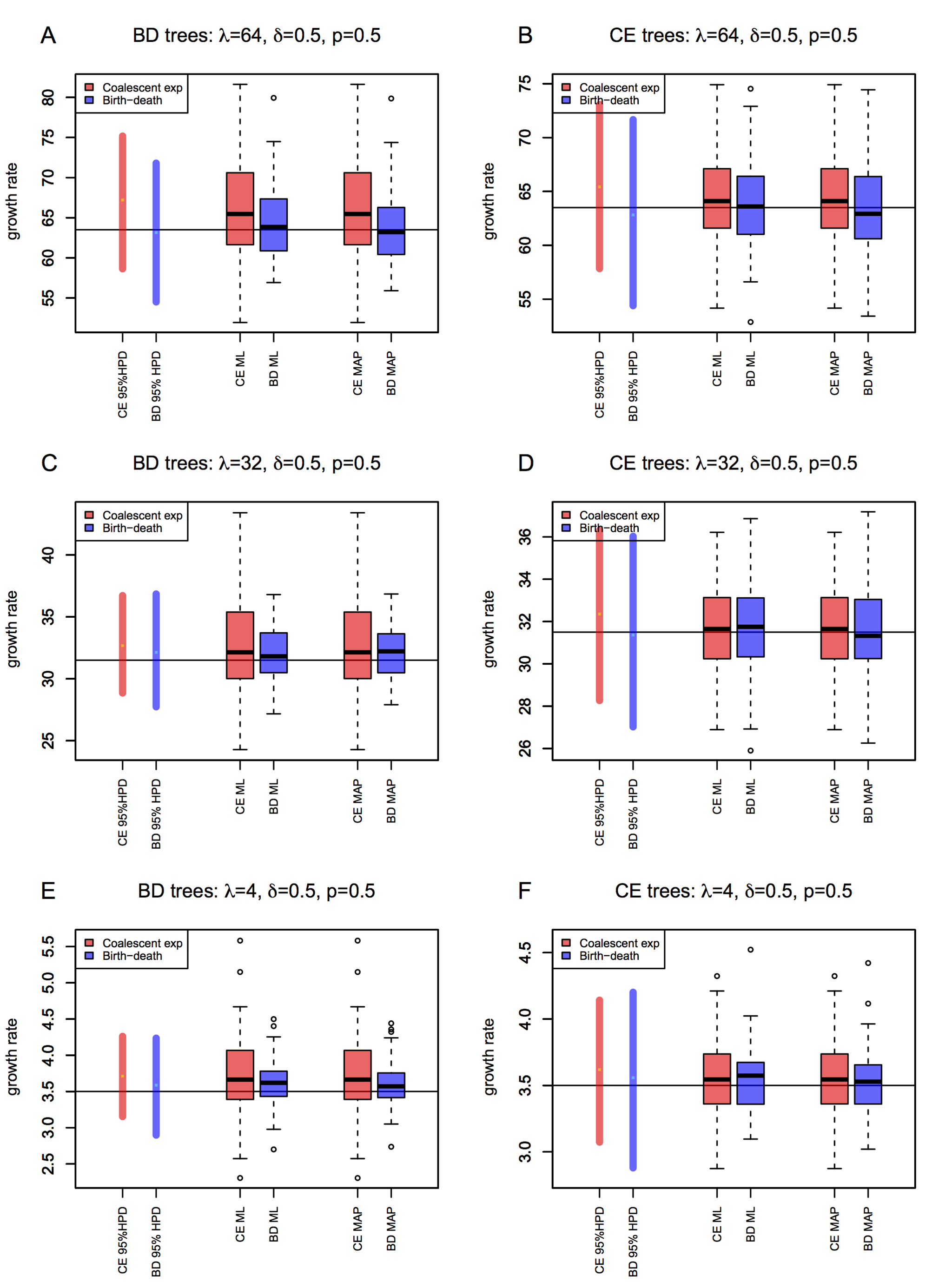

Supplement: Figure S17 — Comparison of growth rate point estimates of the birth-death model and the coalescent model for different values. For and , we display the ML and MAP estimates and the HPD summary for the birth-death trees (A, C, E,…) and the coalescent trees (B, D, F,…). See the legend of Figure 7 for detailed description. (TIF) [file pcbi.1003913.s017.tif]

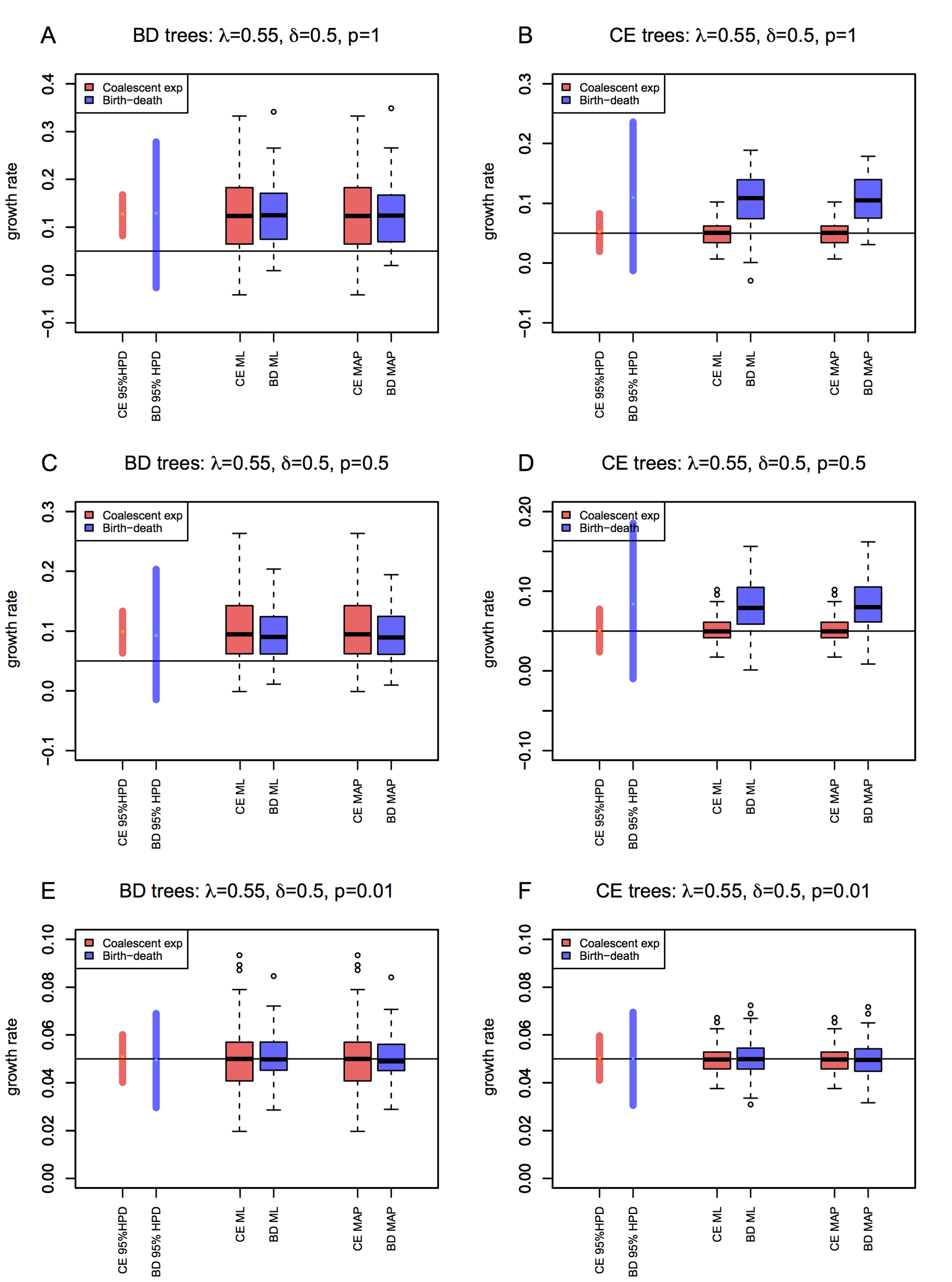

Supplement: Figure S18 — Comparison of growth rate point estimates of the birth-death model and the coalescent model for different sampling probabilities. For and , we display the ML and MAP estimates and the HPD summary for the birth-death trees (A, C, E,…) and the coalescent trees (B, D, F,…). See the legend of Figure 7 for detailed description. (TIF) [file pcbi.1003913.s018.tif]
