# Supplementary material for: Inference of Epidemiological Dynamics Based on Simulated Phylogenies Using Birth-Death and Coalescent Models
Source: PLoS Comput Biol. 2014 Nov 6;10(11):e1003913. doi: 10.1371/journal.pcbi.1003913 (PMC4222655; doi:10.1371/journal.pcbi.1003913)
Supplement: Table S1 — Summary of growth rate parameter estimation statistics at various corresponding to the same . For each of the 100 trees simulated under the respective model (the birth-death or the coalescent), with and various growth rates corresponding to , we estimated the coverage, the 95% HPD interval sizes and RMSE of by the birth-death model and the coalescent model, and display the summary of these measures. Each value of that corresponds to the same growth rate across all the settings is marked in bold. Different simulations corresponding to the same are separated by horizontal double line. (PDF) [file pcbi.1003913.s019.pdf]

Table S1. Summary of growth rate parameter estimation statistics at various $r = \lambda - \delta$ corresponding to the same R_0

	birth-death model trees						coalescent trees					
	birth-death			coalescent			birth-death			coalescent		
	recovered	HPD size	RMSE	recovered	HPD size	RMSE	recovered	HPD size	RMSE	recovered	HPD size	RMSE
$\mathbf{R_0 = 11}, \lambda = 0.55, \delta = 0.05, p = 0.5$	95	0.352	0.084	70	0.305	0.147	98	0.348	0.070	92	0.294	0.081
$R_0 = 2, \lambda = 10, \delta = 5, p = 0.5$	96	0.751	0.199	57	0.453	0.302	99	0.746	0.144	94	0.427	**0.120**
$\mathbf{R_0 = 2}, \lambda = 1, \delta = 0.5, p = 0.5$	92	0.766	0.224	55	0.432	0.332	97	0.752	0.164	92	0.407	**0.113**
$R_0 = 2, \lambda = 0.1, \delta = 0.05, p = 0.5$	92	0.763	0.203	53	0.430	0.343	98	0.744	0.138	95	0.409	**0.103**
$\mathbf{R_0 = 1.14}, \lambda = 4, \delta = 3.5, p = 0.5$	86	3.213	1.040	34	1.117	1.293	87	2.758	0.743	**91**	0.894	**0.284**
$\mathbf{R_0 = 1.1}, \lambda = 5.5, \delta = 5, p = 0.5$	89	4.427	1.387	36	1.353	1.617	86	3.463	0.987	**91**	1.094	**0.351**
$R_0 = 1.1, \lambda = 0.55, \delta = 0.5, p = 0.5$	94	4.363	1.278	28	1.440	1.608	90	3.656	0.914	**93**	1.090	**0.335**
$R_0 = 1.1, \lambda = 0.055, \delta = 0.05, p = 0.5$	96	4.405	1.246	27	1.352	1.628	90	3.757	0.926	**93**	1.084	**0.320**

For each of the 100 trees simulated under the respective model (the birth-death or the coalescent), with $p = 0.5$ and various growth rates $r = \lambda - \delta$ corresponding to $R_0 = \{11, 2, 1.14, 1.1\}$, we estimated the coverage, the 95% HPD interval sizes and RMSE of r by the birth-death model and the coalescent model, and display the summary of these measures. Each value of R_0 that corresponds to the same growth rate $r = 0.5$ across all the R_0 settings is marked in bold. Different simulations corresponding to the same R_0 are separated by horizontal double line.
