# Supplementary material for: Inference of Epidemiological Dynamics Based on Simulated Phylogenies Using Birth-Death and Coalescent Models
Source: PLoS Comput Biol. 2014 Nov 6;10(11):e1003913. doi: 10.1371/journal.pcbi.1003913 (PMC4222655; doi:10.1371/journal.pcbi.1003913)
Supplement: Table S2 — Summary of growth rate parameter estimation statistics in various sampling scenarios. For each of the 100 trees simulated under the respective model (the birth-death or the coalescent), with periods of sampling probability alternating with periods of or , we estimated the coverage, the 95% HPD interval sizes and RMSE of by the birth-death model (1 interval), the birth-death skyline model allowing for varying sampling proportion (10 equidistant intervals for ) and the coalescent model, and display the summary of these measures. Different simulations corresponding to the same are separated by horizontal double line. corresponds to , corresponds to and corresponds to . (PDF) [file pcbi.1003913.s020.pdf]

Table S2. Summary of growth rate parameter estimation statistics in various sampling scenarios

	birth-death model trees						coalescent trees					
	birth-death			coalescent			birth-death			coalescent		
	recovered	HPD size	RMSE	recovered	HPD size	RMSE	recovered	HPD size	RMSE	recovered	HPD size	RMSE
$\mathbf{R_0 = 128}, p = 1$	87	0.284	0.085	62	0.264	0.144	98	0.286	0.065	91	0.251	0.080
$p = 0$ from 0 to 0.15, 10 intervals	59	0.357	0.240	-	-	-	65	0.352	0.193	-	-	-
$p = 0$ from 0 to 0.15, 1 interval	19	0.346	0.412	**69**	0.243	**0.135**	18	0.336	0.365	92	0.237	**0.073**
$p = 0.2$ from 0 to 0.15, 10 intervals	79	0.339	0.141	-	-	-	78	0.338	0.129	-	-	-
$p = 0.2$ from 0 to 0.15, 1 interval	28	0.313	0.231	**76**	0.250	**0.121**	31	0.312	0.219	**90**	0.244	**0.075**
$p = 0$ from 0.04 to 0.16, 10 intervals	39	0.369	0.302	-	-	-	40	0.369	0.265	-	-	-
$p = 0$ from 0.04 to 0.16, 1 interval	8	0.362	0.485	**67**	0.243	**0.124**	6	0.363	0.450	**90**	0.234	**0.070**
$p = 0.2$ from 0.04 to 0.16, 10 intervals	84	0.337	0.127	-	-	-	81	0.336	0.122	-	-	-
$p = 0.2$ from 0.04 to 0.16, 1 interval	41	0.302	0.193	**75**	0.253	**0.115**	44	0.305	0.194	**93**	0.235	**0.063**
$\mathbf{R_0 = 4}, p = 1$	98	0.577	0.150	65	0.384	0.197	98	0.573	0.117	89	0.362	**0.113**
$p = 0$ from 0 to 4, 10 intervals	58	0.543	0.323	-	-	-	61	0.544	0.277	-	-	-
$p = 0$ from 0 to 4, 1 interval	16	0.533	0.708	**75**	0.341	**0.153**	16	0.535	0.660	**95**	0.323	**0.089**
$p = 0.2$ from 0 to 4, 10 intervals	81	0.554	0.220	-	-	-	88	0.535	0.163	-	-	-
$p = 0.2$ from 0 to 4, 1 interval	37	0.503	0.362	**66**	0.349	**0.190**	38	0.504	0.334	**97**	0.331	**0.079**
$p = 0$ from 1.3 to 5.2, 10 intervals	23	0.461	0.387	-	-	-	19	0.458	0.356	-	-	-
$p = 0$ from 1.3 to 5.2, 1 interval	1	0.453	0.711	**73**	0.262	**0.118**	2	0.448	0.677	**94**	0.249	**0.070**
$p = 0.2$ from 1.3 to 5.2, 10 intervals	91	0.498	0.137	-	-	-	97	0.486	0.128	-	-	-
$p = 0.2$ from 1.3 to 5.2, 1 interval	74	0.404	0.174	56	0.318	0.200	77	0.398	0.183	**93**	0.301	**0.089**
$\mathbf{R_0 = 1.1}, p = 1$	89	6.244	2.088	25	1.858	2.400	86	4.716	1.407	**94**	1.416	**0.420**
$p = 0$ from 0 to 30, 10 intervals	67	3.957	1.801	-	-	-	80	3.622	1.300	-	-	-
$p = 0$ from 0 to 30, 1 interval	31	4.241	4.307	**63**	1.887	**1.112**	29	4.016	3.492	**98**	1.664	**0.447**
$p = 0.2$ from 0 to 30, 10 intervals	84	4.012	1.397	-	-	-	96	3.444	0.810	-	-	-
$p = 0.2$ from 0 to 30, 1 interval	59	4.072	2.192	44	1.065	**0.966**	59	3.487	1.677	**91**	0.919	**0.297**
$p = 0$ from 10 to 40, 10 intervals	54	3.139	1.768	-	-	-	72	2.823	1.043	-	-	-
$p = 0$ from 10 to 40, 1 interval	18	3.402	3.392	**29**	0.578	**0.766**	22	3.063	2.457	**98**	0.526	**0.133**
$p = 0.2$ from 10 to 40, 10 intervals	95	3.843	1.163	-	-	-	99	3.395	0.501	-	-	-
$p = 0.2$ from 10 to 40, 1 interval	91	3.675	1.094	36	0.841	1.578	92	3.346	0.865	**93**	0.718	**0.240**

For each of the 100 trees simulated under the respective model (the birth-death or the coalescent), with periods of $p = 1$ sampling probability alternating with periods of $p = 0$ or $p = 0.2$, we estimated the coverage, the 95% HPD interval sizes and RMSE of r by the birth-death model (1 interval), the birth-death skyline model allowing for varying sampling proportion p (10 equidistant intervals for p) and the coalescent model, and display the summary of these measures. Different simulations corresponding to the same R_0 are separated by horizontal double line. $R_0 = 128$ corresponds to $\lambda = 64, \delta = 0.5$, $R_0 = 4$ corresponds to $\lambda = 2, \delta = 0.5$ and $R_0 = 1.1$ corresponds to $\lambda = 0.55, \delta = 0.5$.
