# Supplementary material for: Inference of Epidemiological Dynamics Based on Simulated Phylogenies Using Birth-Death and Coalescent Models
Source: PLoS Comput Biol. 2014 Nov 6;10(11):e1003913. doi: 10.1371/journal.pcbi.1003913 (PMC4222655; doi:10.1371/journal.pcbi.1003913)
Supplement: Table S3 — Summary of growth rate parameter estimation statistics for trees with tips sampled at one point in time. For each of the 100 trees simulated under the birth-death model, with and , we estimated the coverage, the 95% HPD interval sizes and the RMSE of by the birth-death model and the coalescent model, and display the summary of these measures. (PDF) [file pcbi.1003913.s021.pdf]

Table S3. Summary of growth rate parameter estimation statistics for trees with tips sampled at one point in time

	birth-death model trees					
	birth-death			coalescent		
	recovered	HPD size	RMSE	recovered	HPD size	RMSE
$R_0 = 128, \lambda = 64, \delta = 0.5, \rho = 1$	96	0.463	0.122	68	0.461	0.270
$R_0 = 128, \lambda = 64, \delta = 0.5, \rho = 0.5$	94	0.390	0.103	73	0.376	0.183
$R_0 = 128, \lambda = 64, \delta = 0.5, \rho = 0.01$	96	0.164	0.049	75	0.251	0.113
$R_0 = 1.1, \lambda = 0.55, \delta = 0.5, \rho = 1$	95	2.480	0.665	70	1.292	0.728
$R_0 = 1.1, \lambda = 0.55, \delta = 0.5, \rho = 0.5$	96	1.649	0.535	63	0.934	0.581
$R_0 = 1.1, \lambda = 0.55, \delta = 0.5, \rho = 0.01$	91	0.452	0.131	73	0.295	0.140

For each of the 100 trees simulated under the birth-death model, with $\rho = \{1, 0.5, 0.01\}$ and $R_0 = \{128, 1.1\}$, we estimated the coverage, the 95% HPD interval sizes and the RMSE of r by the birth-death model and the coalescent model, and display the summary of these measures.
