# Supplementary material for: Inference of Epidemiological Dynamics Based on Simulated Phylogenies Using Birth-Death and Coalescent Models
Source: PLoS Comput Biol. 2014 Nov 6;10(11):e1003913. doi: 10.1371/journal.pcbi.1003913 (PMC4222655; doi:10.1371/journal.pcbi.1003913)
Supplement: Supplementary Material S1 — First part describes the derivation of waiting times until coalescent under discrete time Wright-Fisher, discrete time Moran, and continuous time Wright-Fisher and Moran population models. Second part discusses parameter correlations under the birth-death process. (PDF) [file pcbi.1003913.s022.pdf]

Inference of epidemiological dynamics based on simulated phylogenies using birth-death and coalescent models

Veronika Boskova[1,*], Sebastian Bonhoeffer[2], Tanja Stadler[1,*]

1 Department of Biosystems Science & Engineering (D-BSSE), Eidgenössische Technische Hochschule (ETH) Zürich, Basel, Switzerland

2 Institute of Integrative Biology, Eidgenössische Technische Hochschule (ETH) Zürich, Zürich, Switzerland

∗ E-mail: veronika.boskova@bsse.ethz.ch, tanja.stadler@bsse.ethz.ch

Supplementary Materials and Methods

Waiting times until coalescent

We derive the waiting times until coalescent events under the continuous time approximation of the discrete-time Wright-Fisher and Moran model. These waiting times are the waiting times of the Kingman coalescent. The approximations require the sample size to be small compared to the population size.

Further, we define a continuous time Wright-Fisher and Moran model and show that the waiting times until coalescent are equivalent to the Kingman coalescent. No assumption about small sample size is required.

Discrete time Wright-Fisher model

Under the Wright-Fisher model, we have discrete generations of I individuals each. Each individual in the daughter generation chooses its parent from the parent generation uniformly at random. Thus, the probability of 2 lineages from the total population of I individuals NOT merging in k generations, with time τ being measured in units of I generations, is:

$$P[T(2) > \tau] = \left(1 - \frac{1}{I}\right)^k = \left(1 - \frac{1}{I}\right)^{I\tau} \xrightarrow[I\to\infty]{} \exp[-\tau],$$

meaning that each 2 lineages merge after Exp(1) waiting time.

Further, the probability that none of the j lineages merges with any other $j-1$ lineage in k generations

is equivalent to the $\binom{j}{2}$ pairs not coalescing in k generations, i.e.

$$P[T(j) > \tau] = \exp\left[-\binom{j}{2}\tau\right]. \tag{1}$$

Discrete time Moran model

Under the Moran model, we again assume a population size of I. In each generation one individual is chosen uniformly at random to die, and a second individual is chosen uniformly at random to replace the dead individual (if the same individual is chosen for death and replacement nothing happens, otherwise a coalescent event occurs). The probability that a single lineage is NOT chosen for death (not replaced) for k generations, with time τ being measured in units of I generations, is:

$$P[S(1) > \tau] = \left(1 - \frac{1}{I}\right)^k = \left(1 - \frac{1}{I}\right)^{I\tau} \xrightarrow[I\to\infty]{} \exp[-\tau].$$

Thus, for $I \to \infty$, each lineage is chosen for death after Exp(1) waiting time. Now, consider j lineages in a population of a total of I lineages. The probability that a lineage x (out of the j lineages) does not coalesce with any other of the $j-1$ lineage for time τ is the probability of k death events of lineage x ($P(k)$ which is a Poisson distribution, i.e. $P(k) = \frac{T^k e^{-T}}{k!}$), times the probability that for each death event, lineage x is NOT merged with any of the $j-1$ lineages $\left(\frac{I-j+1}{I}\right)^k$. Thus, the probability of none of the j lineages merging with each other for time τ is,

$$\begin{aligned} P[T(j) > \tau] &= \left(\sum_{k=0}^{\infty} \frac{\tau^k e^{-\tau}}{k!}\left(\frac{I-j+1}{I}\right)^k\right)^j \\ &= \left(e^{-\tau} e^{\tau\left(\frac{I-j+1}{I}\right)}\right)^j \\ &= \exp\left[-\binom{j}{2}\tau\frac{2}{I}\right]. \end{aligned} \tag{2}$$

Continuous time Wright-Fisher and Moran model

Define the continuous time Wright-Fisher model as a model with I present-day lineages, and each pair coalescing after an Exp(1) distributed waiting time. Define the continuous time Moran model as a model with I present-day lineages, and each lineage dying and being replaced with a random coexisting lineage (including itself) after an Exp(1) distributed waiting time.

Using Equations (1) and (2), the waiting time until a coalescent event within the j lineages again is the waiting time of the Kingman coalescent, i.e.

$$P[T(j) > \tau] = \exp\left[-\binom{j}{2}\tau\right] \qquad \text{and} \qquad P[T(j) > \tau] = \exp\left[-\binom{j}{2}\tau\frac{2}{I}\right].$$

Note here that the derivation of $P[T(j) > \tau]$, i.e. the usage of Equations (1) and (2) does not require $I >> j$. In fact, the derivations above even hold for $I = j$. Recall that for the continuous time approximation of the discrete-time Wright-Fisher and Moran model, the assumption $I >> j$ was required.

Parameter correlations under the birth-death process

Let us denote $r = \lambda - \delta$, and $d = \lambda\delta p$. The probability of a given phylogenetic tree, $f[\mathcal{T}|\lambda, \delta, p, x_0]$, conditioned on the tree height (x_0), does not change when changing sampling proportion p, while keeping r and d constant, as $f[\mathcal{T}|\lambda, \delta, p, x_0]$ depends only on the combined parameters d and r (see Equation (3) in main text, and [1]).

In fact, the probability density $f[\mathcal{T}|\lambda, \delta, p, x_0]$ with parameters λ, δ, p is equal to the probability density of a sampled tree $\mathcal{T}$ with sampling proportion $\hat{p}$ and, birth and death rates:

$$\begin{aligned} \hat{\lambda} &= \frac{r + \sqrt{r^2 + \frac{4d}{\hat{p}}}}{2} \\ \hat{\delta} &= \frac{-r + \sqrt{r^2 + \frac{4d}{\hat{p}}}}{2}. \end{aligned}$$

Note that an increase in p leads to a decrease in λ and δ, if d and r are kept constant. Thus setting the sampling proportion p to a false value will disable us from recovering true λ and δ parameters individually. However, we will always be able to recover the same growth rate parameter $r = \lambda - \delta$, and $\lambda\delta p$, independent of the value to which we fix sampling proportion. Unfortunately, the R_0 estimate

depends on the setting of p,

$$R_0(p) \quad = \quad \frac{\lambda}{\delta} = \frac{p\left(r^2 + \frac{2d}{p} + r\sqrt{r^2 + \frac{4d}{p}}\right)}{2d} \tag{3}$$

We directly observe,

$$\begin{aligned} R_0(0) &= 1 \\ R_0(1) &= 1 + \frac{r^2}{2d} + \frac{r}{2d}\sqrt{r^2 + 4d} \end{aligned}$$

For growth rate $r \geq 0$, we rewrite $R_0(p) = \frac{(pr^2)+2d+\sqrt{(pr^2)^2+4d(pr^2)}}{2d}$ and obtain,

$$\begin{aligned} R_0(p\prime) &< R_0(p) \qquad \text{for } p\prime < p \\ \frac{r^2}{d}p + 1 \leq R_0(p) &< \frac{r^2}{d}p + 2, \end{aligned}$$

meaning R_0 grows monotonically with p, within the interval $[1, 1 + \frac{r^2}{2d} + \frac{r}{2d}\sqrt{r^2 + 4d}]$.

For growth rate $r \leq 0$, we rewrite $R_0(p) = \frac{(pr^2)+2d-\sqrt{(pr^2)^2+4d(pr^2)}}{2d}$. We obtain,

$$\frac{dR_0}{dp} = \frac{r^2}{2d}\left[1 - \frac{2pr^2 + 2(2d)}{2\sqrt{(pr^2)^2 + 2(pr^2)(2d)}}\right] < 0,$$

which leads,

$$R_0(p\prime) \quad > \quad R_0(p) \qquad \text{for } p\prime < p,$$

meaning R_0 increases monotonically with decreasing p, within the interval $[1 + \frac{r^2}{2d} + \frac{r}{2d}\sqrt{r^2 + 4d}, 1]$.

References

1. Stadler T, Kühnert D, Bonhoeffer S, Drummond AJ (2013) Birth–death skyline plot reveals temporal changes of epidemic spread in hiv and hepatitis c virus (hcv). Proceedings of the National Academy of Sciences 110: 228–233.
